# Mediterranean Propolis from the Adriatic Sea Islands as a Source of Natural Antioxidants: Comprehensive Chemical Biodiversity Determined by GC-MS, FTIR-ATR, UHPLC-DAD-QqTOF-MS, DPPH and FRAP Assay

**DOI:** 10.3390/antiox9040337

**Published:** 2020-04-20

**Authors:** Lidija Svečnjak, Zvonimir Marijanović, Piotr Okińczyc, Piotr Marek Kuś, Igor Jerković

**Affiliations:** 1 Faculty of Agriculture, University of Zagreb, Svetošimunska cesta 25, 10000 Zagreb, Croatia; lsvecnjak@agr.hr; 2 Faculty of Chemistry and Technology, University of Split, Ruđera Boškovića 35, 21000 Split, Croatia; zmarijanovic@ktf-split.hr; 3 Department of Pharmacognosy and Herbal Medicines, Wrocław Medical University, ul. Borowska 211a, 50-556 Wrocław, Poland; piotr.okinczyc@umed.wroc.pl

**Keywords:** α-pinene, cadinane type sesquiterpenes, eudesmol isomers, bulnesol and guaiol, clerodane, labdane and abietane diterpenes, methylated flavonoids, esters of phenolic acids, DPPH and FRAP capacity

## Abstract

There is no systematic report about propolis chemical biodiversity from the Adriatic Sea islands affecting its antioxidant capacity. Therefore, the samples from the islands Krk, Rab, Pag, Biševo and Korčula were collected. Comprehensive methods were used to unlock their chemical biodiversity: headspace solid-phase microextraction (HS-SPME) and hydrodistillation (HD) followed by gas chromatography and mass spectrometry (GC-MS); Fourier transform mid-infrared spectroscopy (FT-MIR); ultra high performance liquid chromatography with diode array detector and quadrupole time-of-flight mass spectrometry (UHPLC-DAD-QqTOF-MS) and DPPH and FRAP assay. The volatiles variability enabled differentiation of the samples in 2 groups of Mediterranean propolis: non-poplar type (dominated by α-pinene) and polar type (characterized by cadinane type sesquiterpenes). Spectral variations (FT-MIR) associated with phenolics and other balsam-related components were significant among the samples. The UHPLC profiles allowed to track compounds related to the different botanical sources such as poplar (pinobanksin esters, esters and glycerides of phenolic acids, including prenyl derivatives), coniferous trees (labdane, abietane diterpenes) and *Cistus* spp. (clerodane and labdane diterpenes, methylated myricetin derivatives). The antioxidant potential determined by DPPH ranged 2.6–81.6 mg GAE/g and in FRAP assay 0.1–0.8 mmol Fe^2+^/g. The highest activity was observed for the samples of *Populus* spp. origin. The antioxidant potential and phenolic/flavonoid content was positively, significantly correlated.

## 1. Introduction

*Apis mellifera* L. propolis, known as the bee glue, combines resins collected by the honey bees from different plant organs, and with beeswax that honey bees additionally incorporate. It has been shown that propolis possesses antioxidant, antibacterial, antifungal and antiviral properties, as well as other beneficial biological activities such as anticancer, anti-inflammatory, antiulcer, hepatoprotective, immunostimulating, antidiabetic, etc. [1,2,3,4]. The efficacy of propolis in different in vitro and in vivo protocols suggests its therapeutic properties [1,3]. Reported biological activities have been related to propolis chemical complexity. Propolis has been investigated by utilizing various analytical tools [5] in order to determine its composition, physico-chemical and bioactive properties, as well as specific chemical markers. 

Since the 1960s, numerous studies have revealed propolis composition variability [2,6,7] with more than 300 natural organic compounds: flavonoids, phenolic acids and their esters, polyphenols, terpenes (particularly sesquiterpenes, diterpenes and triterpenes), lignans, steroids, hydrocarbons, amino acids and others. Their abundance has been influenced by botanical and geographical factors, as well as by the season [2,3]. According to the specific chemical composition, different types of propolis are referred in the literature [3]: (a) Poplar type (*Populus* spp.), (b) Birch type (*Betula verrucosa* Ehrh.), (c) Green type (*Baccharis* spp.), (d) Red type (*Dalbergia* spp.), (e) Clusia type (from *Clusia* spp.), (f) Pacific type (*Macaranga tanarius* (L.) Mull.Arg.) and (g) Mediterranean type (mostly from *Cupressaceae/Juniperus* family). Propolis of the European poplar and Brazilian red and/or green (*Baccharis* spp.) types from the continental geographical regions have been widely studied; on the other hand, propolis from more remote locations with specific flora (such as islands), have been investigated only sporadically. There are several records on the island propolis: Pacific propolis from Taiwan, Okinawa and Indonesia islands [8,9,10], and Mediterranean propolis from Malta, Gozo, Cyprus and Greek islands [11]. In last decade, a new type of Greek Mediterranean propolis was reported that contains mainly diterpenes (e.g., communic, cupressic and isocupressic acids and totarol) and almost no phenolics [12].

Due to its specific geographical position on the dividing line between several biogeographic regions and ecological, climatic and geomorphologic conditions, Croatian flora shows high biodiversity with many endemic plants; the ratio of plant species (ca. 10,000 taxa) and territory sets Croatia amongst three European countries richest in flora [13]. Such biodiversity is expected to influence the composition of propolis. Adriatic region of Croatia is populated by a number of islands of which 48 are inhabited. Propolis is regularly being collected from approximately 15 islands as an apicultural by-product. This type of propolis mostly originate from *Cupressus sempervirens* L., *Juniperus* spp., *Pinus halepensis* Miller, *Pinus nigra* J.F.Arnold, *Quercus* ilex L., *Fraxinus ornus* L. and *Olea europaea* L., depending on the vegetation that dominates on specific microlocation/island [5,14].

Research papers on Croatian propolis exist [14,15,16,17,18] and report great variability of Croatian propolis from the continental and Adriatic region. According to the data from available literature, Mediterranean propolis from Croatia has been investigated in several studies [14,18]. However, there is no systematic report about propolis chemical biodiversity from the Adriatic Sea islands as a part of Mediterranean type propolis. Therefore, specific goals of the present study on unlocking Mediterranean propolis from the Adriatic sea islands as source of natural antioxidants are: (a) to select typical propolis from 5 Croatian islands; (b) to isolate volatiles of the samples by headspace solid-phase microextraction (HS-SPME) and hydrodistillation (HD) and to analyze them by gas chromatography and mass spectrometry (GC-MS); (c) to obtain full chemical profile of the samples by Fourier transform mid-infrared spectroscopy (FT-MIR); (d) to determine non-volatile composition of the samples by ultra high performance liquid chromatography with diode array detector and quadrupole time-of-flight mass spectrometry (UHPLC-DAD-QqTOF-MS); (e) to compare the obtained results mutually and with other Mediterranean type propolis; (f) to determine antiradical and total antioxidant activities of the samples by DPPH and FRAP assay.

## 2. Materials and Methods

### 2.1. Sampling of Mediterranean Propolis from the Adriatic Sea Islands

Seven raw proplis samples were obtained directly from the Carniolan honey bee (*Apis mellifera carnica* Pollmann) hives maintained on the islands by the local beekeepers. The samples were collected during 2017 from beehives situated on 5 Croatian islands along the Adriatic Sea coast: Krk (*n = 3* from different island locations; K1P—*Omišalj*, K2P—*Pinezići*, K3P—*Draga Bašćanska*), Rab (RP), Pag (PP), Biševo (BP) and Korčula (KP). The samples were stored in the glass containers in the dark at room temperature, prior to the analyses.

### 2.2. Headspace Solid-Phase Microextraction (HS-SPME) and Hydrodistillation (HD) Followed by Gas Chromatography and Mass Spectrometry Analysis (GC-MS)

HS-SPME was performed with a manual SPME holder using three fibers covered with Polydimethylsiloxane/Divinylbenzene (PDMS/DVB), Carboxen/PDMS (CAR/PDMS) and DVB/CAR/PDMS obtained from Supelco Co. (Bellefonte, PA, USA). For HS-SPME, the finely grinded samples (1 g; manually grinded on a hand grinder) were placed separately in 10 mL glass vials and hermetically sealed. The vials were maintained at 60 °C during equilibration (15 min) and extraction (45 min). Thereafter, the SPME fiber was withdrawn and inserted into GC-MS injector (250 °C) for 6 min for thermal desorption. The procedure was similar as in previous paper [18]. HS-SPME was done in triplicate and the results are presented as mean values.

HD was performed in a modified Clevenger apparatus for 2 h with 1 mL of the solvent trap of the pentane:diethyl ether (1:2 *v*/*v*) and 10 g of the sample cut into small pieces. The volatile oil dissolved in the solvent trap was passed through a layer of MgSO_4_ in a small glass funnel and carefully concentrated by a slow flow of nitrogen, to the volume of 0.2 mL. HD was performed in triplicate and the results are presented as mean value.

Gas chromatography and mass spectrometry (GC-MS) analyses were done on an Agilent Technologies (Palo Alto, CA, USA) gas chromatograph model 7890A equipped with a mass spectrometer (MSD) model 5977E (Palo Alto, CA, USA) and HP-5MS capillary column (5% phenyl-methylpolysiloxane, Agilent J and W). The GC conditions were the same as reported previously [18]. In brief, the oven temperature was set at 70 °C for 2 min, then increased from 70 to 200 °C (3 °C/min) and held at 200 °C for 15 min; the carrier gas was helium (1.0 mL/min). The compounds identification was based on the comparison of their retention indices (RI), determined relatively to the retention times of *n*-alkanes (C_9_–C_25_), with those reported in the literature [19] and those from Wiley 9 (Wiley, New York, NY, USA) and NIST 17 (d-Gaithersburg) mass spectral libraries. The percentage composition of the samples was computed from the GC peak areas using the normalization method (without correction factors).

### 2.3. FTIR-ATR Spectroscopy

Raw propolis samples were analyzed by Fourier transform mid-infrared spectroscopy (FT-MIR) coupled with an Attenuated Total Reflectance (ATR) accessory. Infrared (IR) spectra of collected raw propolis samples were acquired by Cary 660 Fourier transform mid-infrared spectrometer (Agilent Technologies, Palo Alto, CA, USA) coupled with a Golden Gate single-reflection diamond ATR accessory (Specac). Five replicate spectra of each sample (50 scans/spectrum using nominal resolution of 4 cm^−1^) were recorded in the mid-infrared region (4000–400 cm^−1^) using five different aliquots of the sample. An average spectrum was determined for each sample (partial inhomogeneity of raw propolis samples was taken into account in order to obtain representative sample’s spectrum). The sample measurements were recorded at 25 ± 2 °C by using a sapphire self-leveling pressure anvil to create a thin sample layer between the diamond and sapphire for equal spectra acquisition. Approximately 0.05 g of a sample was used to acquire the spectra. Before the acquisition of the following sample spectrum, the ATR diamond and sapphire anvil were cleaned with ethanol (96%) using a soft tissue paper. Raw spectral data were stored and pre-analyzed using the Agilent Resolutions Pro version 5.3.0 (2015) software package (Agilent Technologies, Palo Alto, CA, USA) while further data analysis and chemometric modeling, was carried out using Origin, version 8.1 (Origin Lab Corporation, Northampton, MA, USA).

### 2.4. Preparation of Propolis Extracts for Colorimetric Tests and Liquid Chromatography

An aliquot of 1 g of each propolis sample, was accurately weighted and extracted with 70% ethanol in water, with the ratio 1:10 (g/mL) in an Sonorex Digital 10 P ultrasonic bath (Bandelin, Berlin, Germany). The extraction was conducted for 45 min at 40 °C and the power was set at 90% (576 W). Afterwards, the extracts were centrifuged and filtered through CHROMAFIL^®^ 0.2 µm PTFE filters (Macherey-Nagel, Düren, Germany).

### 2.5. High Performance Liquid Chromatography and Mass Spectrometry (UHPLC-DAD-QqTOF-MS)

UHPLC analyses were performed as previously described [20] with a Thermo Scientific UltiMate 3000 system (Thermo Scientific™ Dionex™, Sunnyvale, CA, USA), coupled with an autosampler and DAD detector recording spectral data in the 200–600 nm range and monitoring at 280, 320 and 360 nm. Chromatographic separation was done using Kinetex^®^ F5 2.6 µm, 100 Å, 150 × 2.1 mm analytical column, equipped with SecurityGuard™ ULTRA F5 guard column (Phenomenex, Torrence, CA, USA) thermostated at 35 °C. The mobile phase consisted of 0.1% formic acid in water (solvent A) or acetonitrile (solvent B). The flow rate was set at 0.4 mL/min and a following gradient program was applied: starting with 100% of solvent A and decreasing to reach 91% at 7 min, held isocratic to 10 min, reaching 80% A at 10.5 min and 60% A at 18.5 min, held isocratic to 22.5 min and decreasing to 0% A at 28.5 min, held isocratic until 32 min. Subsequently, it returned to 100% A and was stabilized for 10 min before the next analysis. The injection volume was 1 µL. Before the analysis, all the extracts were dissolved in ethanol and filtered through CHROMAFIL^®^ 0.2 µm, Ø13 mm, H-PTFE membrane syringe filter (Macherey-Nagel, Düren, Germany). Standard compounds were dissolved in ethanol or mixture of DMSO-ethanol (1:10 *v*/*v*) for hardly soluble compounds and diluted to obtain calibration curves in the range of concentrations 0.5–200 g/mL. Quantitative results were calculated using the calibration curves of appropriate standard or corresponding parent compound (e.g., amount of pinobanksin-3-*O*-acetate was calculated as pinobanksin equivalent taking into consideration the differences in molar mass).

UHPLC-DAD-QqTOF-MS was performed in a similar setting and chromatographic conditions using MS-grade solvents. Compact QqTOF-MS detector (Bruker, Darmstadt, Germany) was used in ESI negative mode, ion source temperature was set at 100 °C, nebulizer gas pressure was set at 2.0 bar, dry gas flow 0.8 L/min and temperature 210 °C. The capillary voltage was set at 2.20 kV and collision energy at 8.0 eV. A 10 mM solution of sodium formate clusters was used for internal calibration. For ESI-MS/MS experiments, collision energy was 35 eV and nitrogen was used as collision gas.

### 2.6. Total Flavonoid (TF), Total Phenolic (TP) Content and Antioxidant Potential (DPPH and FRAP Assays)

#### 2.6.1. Total Antioxidant Activity (FRAP Assay)

The ferric reducing antioxidant assay (FRAP) was performed as previously described [20,21]. Briefly, the reagent was prepared by mixing 10 mmol/L TPTZ reagent (2,4,6-tri(2-pyridyl)-*s*-triazine) with 20 mmol/L ferric chloride in acetate buffer (pH 3.6). The quantitative results were calculated using a calibration curve of ferrous sulfate used as external standard (0.02–1.5 μmol/mL). Before the analysis, the propolis extracts were diluted 20–200 times and 20 μL of the extract solutions were mixed with 200 μL of ferric complex. The results were calculated and expressed as micromoles of Fe^2+^ per gram of propolis. The absorbance (λ = 593 nm) was read in disposable optical polystyrene 96-well plates (FL medical, Torreglia, Italy) using a Multiskan™ GO Microplate Spectrophotometer (Thermo Fisher Scientific, Waltham, MA, USA). All the measurements were performed in triplicate.

#### 2.6.2. Total Phenolic Content (TP)

The total phenolic content was measured spectrophotometrically using the Folin-Ciocalteu method, as previously described [22,23]. Before the analysis, the propolis extracts were diluted 20–200 times and 50 μL of the ethanolic extract solution were mixed with 20 μL of Folin-Ciocalteu reagent. After 5 min, 200 μL of 100 g/L Na_2_CO_3_ solution was added. After 90 min of incubation at room temperature, in dark, the absorbance was read against blank (prepared similarly, using pure solvent instead of sample) at 725 nm in disposable polystyrene 96-well plates using a microplate spectrophotometer. Total phenolic content was calculated using a calibration curve prepared with fresh gallic acid standard solutions (10–200 μg/mL) and expressed as milligrams of gallic acid equivalent (GAE) per gram of propolis. All the measurements were performed in triplicate.

#### 2.6.3. Total Flavonoid Content (TF)

The total flavonoid content was measured spectrophotometrically using a modified pharmacopoeial method with aluminum chloride [24]. An aliquot of 50 μL of prepared extracts was mixed with 50 μL of 2% ethanolic solution of AlCl_3_ (*w*/*v*) and after 60 min of incubation at room temperature, in dark, the absorbance was measured at 420 nm using a microplate reader. Total flavonoid content was calculated using a calibration curve prepared with quercetin standard solutions (20–400 μg/mL) and expressed as milligrams of quercetin equivalent (QE) per gram of propolis. All the measurements were performed in triplicate.

#### 2.6.4. Antiradical Activity (DPPH Test)

Determination of antiradical activity using DPPH radical and comparison with the gallic acid activity was performed using previously modified method [25]. Before the analysis, the propolis extracts were diluted 20–200 times and 20 µL of diluted test extracts were mixed with 200 µL of 0.315 mM DPPH solution in methanol and incubated for 30 min at room temperature, in dark. The absorbance (λ = 517 nm) was read in disposable optical polystyrene 96-well plates using microplate spectrophotometer (as previously). All the measurements were performed in triplicate. The obtained data were calculated from appropriate gallic acid calibration curve (2.0–100 μg/mL) and expressed as gallic acid equivalent antioxidant capacity per gram of propolis (mg GAE/g). 

#### 2.6.5. Statistical Analysis

Statistical analysis was performed for correlation of the antioxidant potential and phenolic/flavonoid content using STATISTICA 64 ver. 13.1 (Dell Inc., Tulsa, OK, USA). Pearson’s product-moment correlation was applied to test relations between the investigated parameters and significance was assessed in two-tailed test at the level of significance *p* < 0.05.

## 3. Results and Discussion

### 3.1. HS-SPME/GC-MS and HD/GC-MS

HS-SPME has been used in last decade for the analysis of propolis headspace (HS) volatile organic compounds (VOCs) as a simple and fast method. To obtain comprehensive HS chemical profiles among samples 3 types of fibers were used. For the isolation of volatile and less-volatile compounds HD with solvent trap was used. VOCs composition is strongly dependent on the extraction method. Striking differences were found between chemical profiles of the same sample obtained by HS-SPME and HD and among the samples. It is known that to produce propolis, bees collect various exudates including balsams, resins and waxes from the plants available in specific areas. It results in different typologies of the final product and therefore the samples were divided (according to VOC results) into two groups depending on the probable plant sources. 

#### 3.1.1. Mediterranean Propolis (Non-Poplar Type)

According to the chemical composition of HS and essential oil (EO) (Table 1 and Table 2), the samples BP and KP were classified in this group. Those two samples were found peculiar as expected, since the islands Biševo and Korčula are more distant from the mainland and are populated by a specific flora.

The most striking difference in the HS composition was found in BP. The predominant HS compound was the cyclic monoterpene hydrocarbon α-pinene (32.9–52.7%) that was present in several other samples and elsewhere (Table 1), but with significantly lower percentages. It was found as the major compound (64.6–77.6%) of HS volatiles in Brazilian and Uruguayan propolis, and represented the 29.4% in Estonian propolis [26]. Other abundant monoterpenes in HS of BP, were biosynthetically derived from α-terpinyl cation or α-pinene: *p*-cymenene (0.9–2.4%), limonene (1.2–1.7%), α-campholenal (1.3–1.6%), *trans*-verbenol (2.2–4.8%), α-terpineol (1.2–1.4%), verbenone (2.3–3.2%) and *trans*-carveol (1.5–2.3%). Similar as in BP HS, α-campholenal, *cis*-verbenol, *trans*-verbenol, verbenone, α-terpineol, nonanal, caryophyllene oxide, β-caryophyllene, β-bourbonene and *cis*-calamenene were found in one propolis sample from Southern Italy as unique HS features in distinction from other Italian propolis of different locations [27]. Sesquiterpenes, biosynthetically derived from farnesyl pyrophosphate (FPP), were present as minor HS constituents, the major ones were β-bourbonene (0.7–1.2%), *trans*-β-caryophyllene (2.3–2.7%), α-muurolene (1.3–2.0%), *cis*-calamenene (0.6–1.1%) and caryophyllene oxide (1.3–1.9%). No eudesmol isomers were present (Table 1). Two lower aliphatic aldehydes were found among relevant constituents: nonanal (2.2–5.1%) and decanal (4.0–7.5%). BP EO contained as the major constituent α-pinene (11.3%), but with ca. 3–5 times lower abundance in comparison with HS. In general, α-pinene has been reported usually as a trace among other dominating volatiles in few European propolis EO [28,29] and tropical propolis EO [30]. In 2006 this compound was identified as the major constituent in Greek propolis EO up to 45.8% indicating new type of European propolis [31]. Other abundant oxygenated monoterpenes were: *trans*-verbenol (2.1%), *cis*-verbenol (1.0%), *trans*-*p*-menth-2-ene-1,8-diol (2.9%), verbenone (2.3%) and *trans*-carveol (1.0%). Several oxygenated sesquiterpenes were found in BP EO: caryophyllene oxide (5.8%), guaiol (1.6%) and manoyl oxide (8.7%). Among them, only caryophyllene oxide was found in HS with ca. 2 times lower percentage. Tricyclic diterpenes, formed biosynthetically from geranylgeranyl pyrophosphate, copalyl diphosphate and sandaracopimarenyl cation, were found only in BP EO: dehydroabietane (3.6%), abietadiene (3.1%), dehydroabietal (2.4%), abietadien-18-al (2.6%) and dehydroabietic acid (1.0%) and methyl isopimarate (1.1%). Higher aliphatic hydrocarbons were also present among major constituents of BP EO: eicosane (2.9%), heneicosane (3.2%), docosane (4.2%) and tricosane (11.6%). The same lower aliphatic aldehydes as in BP HS were found: decanal (1.5%) and nonanal (0.5%).

The major monoterpene of KP HS was α-pinene (7.6–13.5%) followed by minor abundance of limonene and α-campholenal. However, it was not present in KP EO. Among sesquiterpenes, the most abundant were longicyclene (2.6–2.9%), *trans*-β-caryophyllene (2.3–2.9%), caryophyllene oxide (1.2–3.7%) and cedrol (1.0–7.7%). It is interesting to note that cedrol was exclusively found in KP HS and longicyclene only in KP HS and K1P HS. Tricyclic sesquiterpene alcohol α-cedrol and tetracyclic sesqiterpene longicyclene are in general rarely found in propolis. α-Cedrol was found by HS-SPME/GC-MS of Turkish (North Eastern Anatolia) propolis [32] in the range 7.0–15.6%, and in Greek propolis by HD/GC-MS (6.3% [31]). Longicyclene was found in HS of Chinese propolis (9.41% [26]). Aliphatic aldehydes were abundant in KP HS: pentanal (1.4–1.6%), hexanal (1.0–1.9%), heptanal (0.9–1.6%), octanal (1.5–3.2%), nonanal (7.2–16.9%) and decanal (6.2–7.8%). Nonanal and decanal were found with higher percentages in Greek propolis by HD/GC-MS [31] and in the headspace of Turkish (Eastern Anatolia) propolis with minor abundance [32]. Another carbonyl compound was 6-methylhepta-3,5-dien-2-one (3.5–6.8%), previously identified in Taiwanese green propolis EO (12.2%; [33]). 2-Methoxy-*p*-cymene (carvacrol methyl ether) was the only aromatic compound that was present in KP HS (6.0–8.5%). It was found previously in Greek propolis by HD/GC-MS (0.4–1.5% [31]) and in traces in Portugese propolis by HD/GC-MS [34]. Another aromatic compound was benzaldehyde (0.5–2.7%). KP EO is quite different in comparison with KP HS containing higher alkanes as the major constituents: heneicosane (4.1%), docosane (13.5%), tricosane (31.8%) and tetracosane (25.4%). Indian propolis EO contained [35] among major constituents long-chain alkanes (tricosane (13.6%), hexacosane, heptacosane and heneicosane). Two sesquiterpene alcohols were found as major compounds in KP EO: guaiol (3.1%) and manool (5.7%). They were found in EO of Greek propolis [31] with relevant percentages (guaiol up to 5% and manool up to 5.2%). 

#### 3.1.2. Comparison with Probable Plant Source Volatiles

*Cupressus* spp. and *Juniperus* spp. have been most frequently reported in last decade as sources of Mediterranean type propolis [27,31] and those plants are naturally widespread, among others parts of the Adriatic region, on the islands Biševo and Korčula as reported in the Flora Croatica Database [36]. α-Pinene found in the investigated samples could originate from exudates of *Cupressus sempervirens* L. known as one of the source plants utilized by the bees to form propolis [27,31] and it is known that EO of *C. sempervirens* from Croatia [37] contains α-pinene as the main component (up to 79.2%). In propolis samples from Southern Italy (Adriatic coast) and Greece α-pinene was also identified at high percentage and other coniferous species were also suggested as the plant source [27,31]. However, the abundance of monoterpene fraction, with a high α-pinene content, was also described for the species of the genus *Juniperus* [38] and it was reported that monoterpenes may also contribute to propolis in specific geographical locations [34]. α-Pinene is found as the main component of the needles EO (41.37%) and berries EO (66.30%; 61,21%) of the wild Croatian *Juniperus oxycedrus* L. [39]. However, the contribution of other plant sources is also possible, especially *Pinus* spp. that are well known to contain α-pinene in the resin EO (21.39–25.40% [40]) and in the headspace (66.2%; 73.4% [41]). Limonene, found with minor abundance in VP HS and EO, was identified in the EO of *C. sempervirens* and *J. oxycedrus* from Croatia [37,38,39] and *Pinus* spp. resin [40,41]. Manoyl oxide (12.29%) and α-campholene aldehyde (0.15%) were present in *J. oxycedrus* needles EO from Croatia [39], in *Pinus* spp. resin (0.4–0.9% [41]) and in BP EO. Manoyl oxide was also identified in *C. sempervirens* EO [37,38]. Manool and guaiol present in KP EO were also found in *Cupressus* and *Juniperus* plants [42,43]. Tricyclic diterpenes (particularly methyl isopimarate, dehydroabietic acid, dehydroabietane, dehydroabietal) were found in *Juniperus* plants [43]. Abietane diterpenoids from *C. sempervirens* were also reported [44] and dehydroabietane was isolated from the cypress EO [45] and in BP EO. α-Cedrol, having a woody and spicy characteristic smell, was found in *C. sempervirens* EO at 23.68% [45] and in the range 1.2–12.9% [37]. Although it was not found in *J. oxycedrus* from Croatia [39] it was found in *J. oxycedrus* from Turkey (2.3%–9.7% [44]). Some of the volatile compounds in BP EO may be linked to other *Cistus* species—for example *Cistus salvifolius* L. and their chemotypes, that provide high volatiles diversity and are dominated by oxygenated sesquiterpenes and monoterpenes [46]. Manoyl oxide is also one of the major constituents of BP EO, but was also the main component of essential oil obtained from *Cistus creticus* L. [47], while essential oil of *C. creticus* subsp. *eriocephalus* was characterized by *i.a.* manoyl oxide, α- and δ-cadinene, viridiflorol and bulnesol [48]. On the other hand, essential oil of other *Cistus* species cultivated in Corsica, such as *Cistus ladaniferus* L. was dominated by α-pinene (11.1–47.4%), that is another relevant compound found in BP EO [49]. In general, the size of *n*-alkanes from black pine needles wax ranged from C_16_ to C_33_ and the most abundant were C_23_, C_25_, C_27_ and C_29_ [50]. *Cupressaceae* leaf wax has been characterized (chemotaxonomic significance) by moderate percentages of *n*-alkanes [51], particularly of C_31_, C_33_, C_27_ and C_21_, including C_22_, C_23_ and C_24_. Heneicosane, docosane, tricosane and tetracosane were found as major constituents in KP EO. Heneicosane was found with very high percentages only in *C. sempervirens* [52]. In addition, all leaf-wax samples of *J. communis* showed predominance of *n*-alkane C_33_ in the needle wax (30.0–61.4%), which appears to be a common feature for *Juniperus* species [53] (the range of *n*-alkanes reported by different authors varied from mid-length (C_23_) to long-chain *n*-alkanes (C_25_–C_35_)). 

#### 3.1.3. Mediterranean Propolis (Poplar Type) Volatiles

According to the chemical composition (Table 1 and Table 2), the samples RP, PP, K1P, K2P and K3P were classified in this group. The islands Rab, Pag and Krk are located closer to the Adriatic coast, characterized by an abundance of *Populus* spp. [36].

Sesquiterpenes represent the most abundant group of compounds in RP HS being dominated by the bicyclic hydrocarbons of cadinane type: δ-cadinene (15.0–21.5%) and γ-cadinene (6.6–9.3%) followed by α-cadinene (1.2–2.1%), α-muurolene (4.4–5.9%), α-copaene (1.9–3.3%) and α-amorphene (2.3–3.5%). Higher percentages of cadinene isomers were previously found as abundant in Albanian propolis EO [54] and HS and EO of northern Italian propolis [27]. Two oxygenated sesquiterpene isomeric alcohols of the selinane series were identified: β-eudesmol (0.6–3.7%) and α-eudesmol (0.6–3.3%). Another group of present constituents was non-terpene aromatic compounds: benzaldehyde (0.2–2.1%), benzyl alcohol (2.1–6.9%), 2-phenylethanol (2.8–6.7%), benzyl acetate (1.2–2.5%), benzoic acid (4.2–14.5%) and phenethyl acetate (0.6–1.2%). Lower aliphatic C_5_-compounds were identified in RP HS (not found in RP EO), the major ones were 3-methylbut-3-en-1-ol (0.1–2.1%), 3-methylbut-2-en-1-ol (0.7–2.7%), 3-methylbut-2-enal (0.2–1.2%) and 2-methylbut-2-enoic acid (1.2–5.7%). γ-Cadinene (3.1%) and δ-cadinene (6.6%) were present in RP EO as well as γ-eudesmol (5.5%), β-eudesmol (9.6%) and α-eudesmol (9.4%). Isomers of cadinene were present with lower abundance in comparison to RP HS. Sesquiterpene alcohols exclusively found in RP EO were: guaiol (4.4%), α-cadinol (10.1%), α-muurolol (1.9%) and bulnesol (2.5%). High abundance of α-cadinol can be pointed as a distinctive characteristic of RP EO. Higher alkanes were also present: heneicosane (1.0%), docosane (11.2%), tricosane (5.2%) and tetracosane (9.4%). EO of Indian propolis [35] was shown to contain 45.83% of long-chain alkanes (including heneicosane, tricosane, hexacosane and heptacosane).

PP HS is quite distinct from other samples, containing two isomeric tertiary bicyclic alcohols with a structure of azulene type guaiol (14.3–28.9%) and bulnesol (7.7–16.7%) as predominant components. They are biosynthetically derived from guaiyl cation and germacryl cation derived from (*E*,*E*)-farnesyl cation. Three typical sesquiterpene alcohols were present: γ-eudesmol (1.6–3.7%), β-eudesmol (3.1–7.3%) and α-eudesmol (1.9–4.7%). The most abundant monterpene in PP HS was α-pinene (3.6–5.9%). Relatively high content of lower aliphatic C_5_-compounds was found in PP HS: 3-methylbut-3-en-1-ol (0.5–1.7%), 2-methylbut-2-enal (0.3–3.2%), 3-methylbut-2-en-1-ol (0.8–2.6%), 3-methylbut-2-enal (0.5–7.9%), 2-methylbutanoic acid (1.2–1.9%) and 2-methylbut-2-enoic acid (0.9%–3.7%). They belong to hemiterpene compounds derived from 3,3-dimethylallyl pyrophosphate and isopentenyl pyrophosphate [55]. Several aromatic compounds were present as minor constituents: benzaldehyde (0.2–2.1%), benzyl alcohol (0.5–1.5%) and 2-phenylethanol (2.1–6.1%). The composition of PP EO is quite peculiar. Guaiol (14.3%) and bulnesol (15.9%) were also found among major PP EO constituents (similar as in PP HS) followed by γ-eudesmol (3.9%), β-eudesmol (6.4%) and α-eudesmol (4.5%). Higher alkanes were predominant in PP EO: heneicosane (3.5%), docosane (12.7%) and tricosane (22.3%). Guaiol was found as major constituent in the extract of French propolis [56] and in the sample of Greek propolis [31]. Bulnesol was found in the extract of Lebanese propolis [57].

In distinction from other samples, acetic acid was abundant in K1P, K2P and K3P HS up to 31.9%. It was already found as the most abundant compound in Chinese propolis (Heilongjiang) HS, accounting for about 60% of the total GC area [58]. It is interesting to note high abundance of monoterpene hydrocarbon limonene, particularly in K2P HS (5.7–11.8%). High content of limonene was found in Croatian propolis EO (10.5%; 11.2% [28]) and Uruguayan propolis HS (15.6%; [26]). α-Longipinene (1.4%; 2.3%; 2.3%) and longicyclene (2.0%; 3.9%; 6.1%) were present only in K1P HS. Benzene derivatives were abundant: benzaldehyde in K1P HS (1.9–9.8%), K2P HS (3.2–17.9%) and K3P HS (2.6–4.7%), benzyl alcohol in K1P HS (1.7–3.7%), 2-phenylethanol in K1P HS (5.8–12.2%) and K3P HS (3.1–5.3%), benzoic acid in K2P HS (13.0–29.2%). Lower aldehydes were present: nonanal in K1P HS (3.2–3.6%), K2P HS (6.1–11.0%) and K3P HS (1.9–5.8%) and decanal in K1P HS (2.2–5.5%), K2P HS (4.1–7.9%) and K3P HS (0.9–3.3%). Guaiol was present in K1P HS (3.2–8.9%) and K3P HS (2.3–2.9%). Aliphatic C_5_-compounds were found: 3-methylbut-3-en-1-ol in K1P HS (0.7–1.7%) and K3P HS (1.7–1.8%), 3-methylbut-2-en-1-ol in K1P HS (1.5–3.7%) and K3P HS (1.5–2.9%), 3-methylbut-2-enal in K3P HS (1.1–4.5%) and 2-methylbutanoic acid in K1P HS (2.0–2.2%) and K3P HS (0.7–1.4%). Thymol was the most abundant in K3P HS (10.1–39.9%). However, thymol is not a typical propolis constituent and can be connected with anti-*Varroa* treatment [18,59]. Typical sesquiterpenes were found in EO (K1P; K2P; K3P): α-eudesmol (0.3–2.6%), β-eudesmol (0.5–5.1%), γ-eudesmol (0.3–2.6%), guaiol (0.0–5.2%) and α-cadinol (0.5–0.8%). Benzyl benzoate was most abundant in K2P EO (13.8%) as well as benzyl cinnamate (14.9%) and benzyl salicylate (2.7%). Several higher alkanes were found in EO (K1P; K2P; K3P): heneicosane (2.2–3.7%), docosane (2.7–23.5%) and tricosane (24.8–35.1%). 

#### 3.1.4. Comparison with *Populus* spp. Volatiles

*Poplar* spp. (*Populus nigra* L., *Populus tremula* L. and *Populus alba* L.) and the buds resin have been reported as a primary source of propolis from temperate zones [6]. As reported in the Flora Croatica Database [36], the area of the islands Pag, Rab and Krk is within the range of different *Poplar* ssp. abundance (particularly *P. nigra*, *P. tremula* and *P. alba*). Black poplar (*P. nigra*) buds exhibited different EO profiles (both qualitatively and quantitatively). Some buds contained mainly oxygenated sesquiterpenes, particularly α-, β- and γ-eudesmols that are present in *P. nigra* buds EO [60,61] as well as their CO_2_ extracts [20]. These compounds are present in K1P, K2P and K3P EO, PP HS and EO as well as RP HS and EO that could be connected with the *P. nigra* (eudesmol chemotype) distribution on the islands Krk, Rab and Pag. *P. nigra* buds EO [20] contained bulnesol (4.4%) and guaiol (5.7%), as well as their supercritical CO_2_ extracts (guaiol (2.7–3.7%); bulnesol (2.5–3.4%)). Hexane extracts of *P. nigra* buds [62] analyzed by GC-MS contained, among other constituents, guaiol (8.7%) and bulnesol (3.8%). Isomers guaiol and bulnesol were characteristic for PP HS and EO as well as RP EO indicating dominant influence of *P. nigra* (bulnesol/guaiol chemotype) from the islands Pag and Rab. Several *P. nigra* buds EO [60] were mainly composed of sesquiterpene hydrocarbons (mainly ar- and γ-curcumene and δ-cadinene). Isomers δ-, γ- and α-cadinene were typical for RP HS and EO that can be also connected with *P. nigra* (cadinene chemotype) distribution on the island Rab. *P. nigra* buds EO was reported also with different profiles containing a mix of sesquiterpenes and derivates of benzoic acid, mainly prenyl benzoate [60]. About 50% of the GC chromatograms of hexane extracts of *P. nigra* buds [62] consisted of higher alkanes including docosane and tricosane (found in RP, PP, K1P, K2P and K3P EO), but C_25_-C_31_ alkanes dominated (they were not present in investigated propolis EO probably due to lower volatility). Higher alkanes are known to be one of the main components of cuticular waxes of plant leaves and stems. Aliphatic alcohols 2-methylbut-3-en-2-ol, 3-methylbutan-1-ol, (*E*)-2-methylbut-2-en-1-ol and (*E*)-2-metylbut-2-enoic acid were identified in *P. nigra* buds EO [61]. These hemiterpenes were present in total amount up to 8%. Hemiterpenes were present in RP HS, PP HS, K1P HS and K3P HS. The esters of hemiterpene (prenyl) alcohols and *cis*/*trans* caffeic, ferulic and isoferulic acids were previously identified in the bud exudate of *P. nigra* [63], but as non-volatile compounds they cannot be isolated by hydrodistillation. Aspen buds (*P. tremula*) also exhibited different EO profiles. Several aspen buds [60] contained mostly benzoic acid derivates (benzyl benzoate, salicyl benzoate and *trans*-benzyl cinnamate). They were identified in K2P EO indicating aspen (*P. tremula*)-type propolis and *P. tremula* is reported on the island Krk [36].

### 3.2. Chemical Characterization by FTIR-ATR Spectroscopy

In most of the FTIR spectroscopic studies, propolis research has been focused on ethanolic propolis extracts (EPE) [64,65,66,67,68,69] while raw beehive propolis that serves as a source (raw material) for preparing propolis-based products (such as the most commonly used propolis ethanolic tincture) has been covered only by two reports [70,71].

Complexity of FTIR-ATR spectrum of raw propolis arises from its complex chemical composition that varies significantly depending on the source of the plant exudate which bees have collected. Still, chemical composition of propolis has generally been represented by two groups of constituents: balsam content (40–70%) mostly comprised of numerous phenolics, and non-balsam content containing beeswax (20–35%), essential oil (3–5%; mono and sesquiterpenes) and other organic compounds (ca. 5%; ash content, polysaccharides: proteins, amino acids, mechanical impurities, etc.). Balsam content is the most complex compositional segment of propolis and includes the following substances: phenols, phenolic acids, esters, flavanones, dihydroflavanones, flavons, flavonols, chalkones, phenolic glycerides and other minor compounds [72].

Given that FTIR spectrum of propolis reflects its overall chemical composition, identification of absorption bands, i.e., assignment of functional groups within the IR spectrum of raw propolis material represents a demanding task due to a large number of various organic compounds and corresponding molecular vibrations that can be observed in it. Nevertheless, it is possible to distinguish signals that are highly specific for particular organic compound based on the comprehensive literature data on propolis chemical composition, as well as various sources of FTIR spectral data (e.g., spectral libraries and atlases).

**Figure 1:**
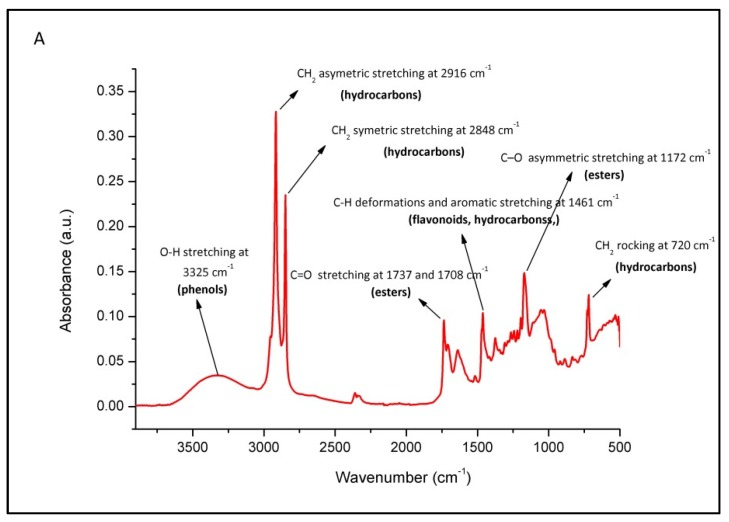

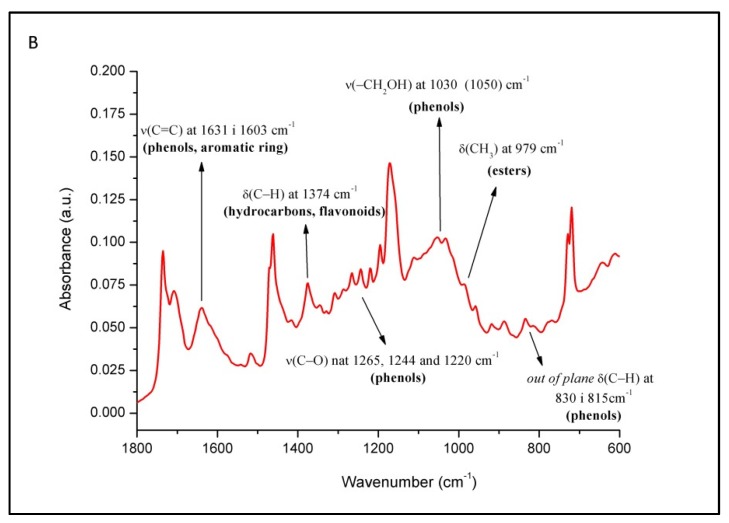
Characteristic FTIR-ATR spectrum of raw propolis (average K3 sample) with assigned underlying molecular vibrations: Spectral region from 3800 to 500 cm^−1^ (**A**); fingerprint region 1800–600 cm^−1^ (**B**).

General assignment of molecular vibrations in the propolis spectrum is presented on an average FTIR-ATR spectrum of K3 sample from Krk island (Figure 1). The complexity of its absorptions is arising from a complex composition dominated by substances from the balsam group of compounds. A broad strong band at 3350 cm^−1^ observed in analyzed propolis samples occurs due to the O–H stretching vibration of the phenolic group. Spectral features related to phenols are also characterized by interaction of O–H deformation and C–O stretching vibrations which can be observed in the spectral range between 1405 and 1220 cm^−1^ (with maximum absorbance at 1375 cm^−1^) and in the form of series of weak vibrations between 1260–1180 cm^−1^. Phenols are also represented with a doublet at 1640 cm^−1^ assigned to aromatic ring C=C stretching and aromatic C–H deformation vibration at 1110 cm^−1^ [73]. A medium absorption at 720 cm^−1^ is peaking due to CH_2_ rocking of hydrocarbons originating from beeswax [72]. An overlapping effect with out-of-plane deformation of the O–H group of phenols is possible in this region. A weak band peaking at 1515 cm^−1^ can be assigned to flavonoids; C=C (aromatic ring) stretching [68]. C–H deformations and aromatic stretching at 1461 cm^−1^ is assigned to flavonoids (hydrocarbons CH_3_ and CH_2_ vibrations are overlapping). The most prominent absorption in the fingerprint region is a broad band with absorption maximum observed at 1170 cm^−1^ that corresponds to the C–O asymmetric stretching vibration of esters related to long-chain aliphatic acids. Saturated aliphatic esters typically absorb at 1750–1725 cm^−1^ [73]. Thus, absorption occurring at 1736 cm^−1^ is due to the carbonyl group (C=O) stretching vibrations of the ester bond. This vibration can be attributed to the monoesters from beeswax in propolis, as the major ester component of beeswax (~40%) [74]. As shown in Figure 1B, other medium and weak intensity absorption bands are attributed to the vibrations of various functional groups of phenols, flavonoids and hydrocarbons, some of which overlap. 

**Figure 2:**
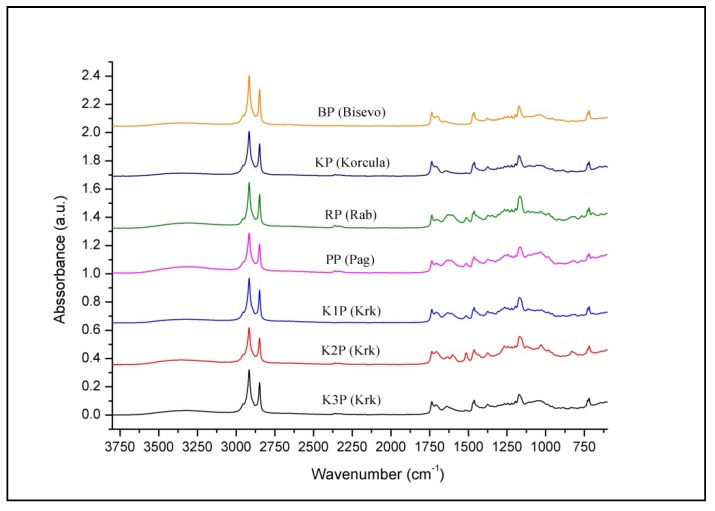
Average FTIR ATR spectrum of investigated propolis collected from five Adriatic Sea islands (Krk, Rab, Pag, Biševo and Korčula).

**Figure 3:**
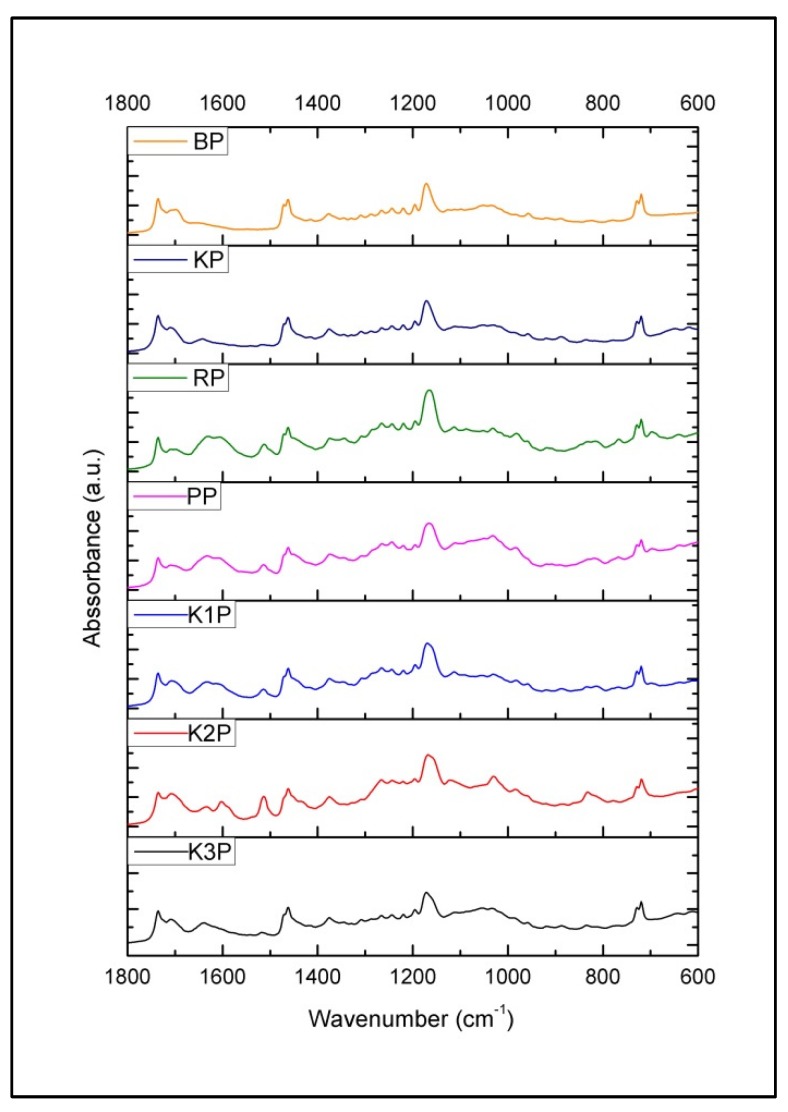
Average FTIR ATR spectrum of investigated propolis collected from five Adriatic Sea islands (Krk, Rab, Pag, Biševo and Korčula)—fingerprint region (1800–600 cm^−1^) emphasizing the most significant spectral variations.

**Figure 4:**
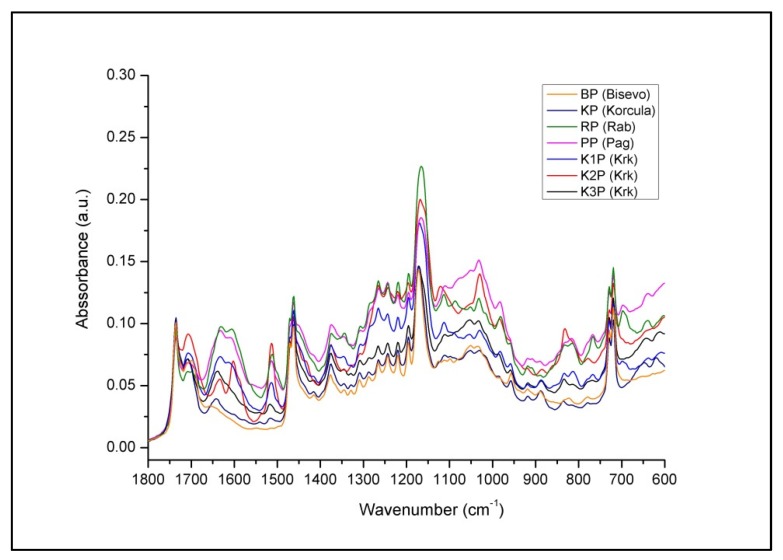
Overlaid average FTIR ATR spectrum of investigated propolis collected from five Adriatic Sea islands (Krk, Rab, Pag, Biševo and Korčula)—fingerprint region (1800–600 cm^−1^) emphasizing comparative spectral variations.

As presented in Figure 2, unique spectral patterns of propolis from different Adriatic Sea islands reflect compositional differences (different band positions and intensities) between the samples and indicate significant compositional differences. Variations in hydrocarbon content (at 2916, 2848, 1461, 730 and 720 cm^−1^) and esters (at 1736 cm^−1^) originating from beeswax present in propolis [70,73] are not distinguished significantly between analyzed propolis samples, as opposed to spectral variations associated with phenolics and other balsam-related components that are clearly observable. These differences are mainly related to the content of phenols, flavonoids and esters, and corresponding spectral variations are most prominent in the fingerprint region (1800–600 cm^−1^). The results of spectral analysis revealed great similarity of propolis samples from the islands Biševo and Korčula indicating similar botanical origin. Two propolis samples from Krk (K1P and K3P) were also found to be similar, while propolis from Pag, Rab, as well as K3P from Krk, showed specificities due to characteristic phenolic and ester bands (indicating that propolis was collected from different resin sources). As presented in Figure 3 and Figure 4, fingerprint region displays a series of multiple absorption bands occurring due to mentioned groups of organic compounds. It can be observed that propolis from Biševo and Korčula exhibit similar spectral pattern in this region, while propolis from other islands (Pag, Rab, Krk) reflect unique spectral features. Among them, Pag propolis and K2P propolis (Krk propolis from Pinezići) are the most distinguished ones due to the high phenolic content (represented by the most prominent phenolic band at 1030 cm^−1^), while Rab (RP) and K2P propolis stand out for their higher ester content (absorption maximum at 1070 cm^−1^).

### 3.3. UHPLC-DAD-QqTOF-MS

The ethanolic extracts of seven propolis samples were analyzed, disclosing high diversity between the samples collected from different Croatian islands. Nearly 120 compounds were identified or tentatively identified in the samples (mainly derivatives of phenolic acids, flavonoids and terpenes (Table 3). Selected major phenolics were quantified and significant differences in their abundance were found. Content of phenolics in RP was much higher than in other samples (Table 4).

The most relevant groups of compounds determined by UHPLC-DAD-QqTOF-MS were phenolics, including flavonoids, phenylpropanoids and simple phenols. The extracts were rich in variety of flavonoids, including numerous methylated derivatives, represented mainly by flavones: chrysin, chysin 5-methyl ether, apigenin, luteolin, luteolin 5-methyl ether; flavanones: pinocembrin, pinocembrin 7-methyl ether (pinostrobin), naringenin, naringenin 7-methyl ether (sakuranetin), eriodyctiol, 4′-methoxy eriodictyol (hesperetin); flavonols: quercetin, quercetin 3-methyl ether, quercetin 7-methyl ether (rhamnetin), quercetin 3′-methyl ether (isorhamnetin), isomers of quercetin dimethyl ether, quercetin 3,7,4′-trimethylether, kaempferol, kaempferol 4′-methyl ether (kaempferide), kaempferol 3,4′-dimethyl ether, pinobanksin, pinobanksin 5-methyl ether, pinobanksin 7-methyl ether, galangin, galangin 5-methyl ether and only in BP: myricetin 7,4′-dimethylether, myricetin 3,7,4′-trimethylether, myricetin 3,7,4′,5′-tetramethylether. Most of those compounds were present in majority of the samples at least in traces. 

The amounts of the most common, quantified compounds determined in all the samples were 0.05–1.76 mg/g (isorhamnetin), 0.24–12.28 mg/g (luteolin-5-*O*-methyl ether), 0.08–1.94 mg/g (rhamnetin; in BP it was found in traces), 0.04–30.71 mg/g (chrysin), 1.70–17.08 mg/g (sakuranetin), 0.13–3.01 mg/g (kaempferol). Elevated abundance of pinobanksin, chrysin, pinocembrin, pinobanksin-3-*O*-acetate, pinobanksin-3-*O*-propanoate were particularly pronounced in RP, K1P, and K3P. Myrycetin 3,7,4′,5′-tetramethyl ether (4.29 mg/g) was found only in BP. The particularly high amounts of different flavonoids were found in RP. The MS data for particular, rarely occurring flavonoid myricetin 3,7,4′,5′-tetramethyl ether were consistent with those previously reported. The exact mass 375.1088 (ESI^+^) corresponded to [M + H^+^]^+^ and the MS/MS spectrum obtained at 35 eV was (*m/z*): 360, 345, 332, 331, 330, 318, 317, 315, 314 [75].

Phenylpropanoids were found both as free caffeic (0.15–6.80 mg/g, BP, KP—traces), *p*-coumaric (0.17–4.22 mg/g, KP—traces), ferulic (0.31–4.10 mg/g, KP—traces) and isoferulic acids (0.09–8.30 mg/g, BP—traces, KP—not detected) but also their derivatives including variety of glyceryl and prenyl esters. The latter were quantified in RP, PP, K1P, K2P, K3P but found as traces in other samples. Other compounds included benzyl esters, cinnamyl esters of caffeic, *p*-coumaric and ferulic acids. Only KP did not contain these compounds (or only traces were found).

UHPLC-QqTOF-MS analyses allowed also to tentatively identify a number of terpenic compounds in the extracts. Among terpenic compounds—labdane type diterpenes: cupressic and isocupressic acid (samples KP, BP); abietane type diterpenoids: dehydroabietic (BP, KP, PP, K1P, K2P), abietic and pimaric acid isomers (BP, KP, traces in some other samples). The MS data for cuppressic acid and isocupressic acid were consistent with literature data [75]. For isocupressic acid the exact mass 321.2445 and 303.2332 (ESI^+^) corresponded to [M + H^+^]^+^ and [M − H_2_O + H^+^]^+^, respectively and the MS/MS spectrum obtained at 35 eV was (*m/z*): 285, 257, 247, 215, 201, 193, 187, 175, 161, 147 and 133. Other diterpenes were found only in BP: 8-hydroxylabdan-15-oic acid (labdane type), 18-hydroxy-*cis*-clerodan-3-ene-15-oic acid and 18-acetoxy-*cis*-clerodan-3-ene-15-oic acid (clerodane type). The MS data for 18-hydroxy-*cis*-clerodan-3-ene-15-oic acid was consistent with the literature data. The exact mass 305.2477 (ESI^+^) corresponded to [M − H_2_O + H^+^]^+^ and the MS/MS spectrum obtained at 35 eV was (*m/z*): 287, 269, 263, 249, 235, 223, 221, 209, 195, 191, 177, 175, 163, 149, 135, 121 and 107 [75]. The MS data for 18-acetoxy-*cis*-clerodan-3-ene-15-oic acid was: 363.2544 (ESI^−^) and the fragments (obtained by MS/MS at 35 eV) 321 (322), and 303 corresponded respectively to the loss of acetyl group and H_2_O. The main pseudomolecular ions in positive ionization 387.2521, and 305.2493 (ESI^+^) corresponded to [M + Na^+^]^+^ and [(M + H)-CH_3_CO-H_2_O + H^+^]^+^. The MS/MS spectrum of the latter fragment obtained at 35 eV was consistent with data for 18-hydroxy-*cis*-clerodan-3-ene-15-oic acid containing (*m/z*): 287, 269, 263, 249, 235, 223, 209, 195, 177, 163, 149, 135, 121 and 107 [75]. Other tentatively identified compounds include triterpenes: oleanoic, moronic (oleanane type) and iso-/masticadienoic acids (euphane type) found in BP, KP, PP, K2P, K3P.

The major compounds reported in most samples of Croatian propolis were phenolic acids (ferulic, *p*-coumaric acid) and flavonoids (galangin, pinocembrin, chrysin) [16,18,76,77,78]. These findings are very similar to the profiles of RP, K1P and K3P from the current study. However, Croatian samples that did not contain these compounds were also reported, demonstrating occurrence of different propolis types in Croatia [16,18]. Saftić et al. recently reported LC-MS analysis of propolis from different regions of Croatia, including Mediterranean samples. The latter contained diterpenes, e.g., pimaric acid, isocupressic acid (found also in some of the currently analyzed samples) but on the other hand also totarol, agathadiol and artepillin C (corresponding exact masses not found in the currently analyzed samples) [15]. These compounds were previously proposed as markers for Mediterranean propolis poor in flavonoids and phenolic acids, deriving mainly from *Cupressus* spp. [5].

#### Possible Botanical Origin of the Samples Based on LC-MS Profiles

All the samples contained at least traces of prenyl caffeates, recognized as typical for the most common European black poplar-type propolis [60], however their total amount was more relevant only in three samples RP, K1P and K3P (37.74, 11.49, 5.92 mg/g, respectively), demonstrating primary (RP) or secondary (K1P and K3P) contribution of *P. nigra* balsam in those specimens (Table 4). The same samples contained also another typical compounds from this source, such as pinobanksin 3-*O*-acetate, chrysin, pinocembrin and others [60] which again were very abundant in RP. RP contained particularly high amount of isoferulic acid (8.30 mg/g), while K1P and K3P only its slightly elevated amount (0.67–0.80 mg/g); though the amount of isoferulic acid was 2–4 times higher than of ferulic acid, which suggests that the bud exudate was partially collected from *P. nigra* var. *italica* Münchh. [79]. Different profiles of other poplar polyphenols were described in literature [60,79]. The highest differences between various *Populus* species were connected to the presence of various flavonoids, presence of both flavonoids and phenolic acids as well as their monoesters, or the presence of mainly free phenolic acids. Sakuranetin was found in both *P. nigra* [60,80] and *P. tremula* [60]. This may partially explain the origin of higher amounts of this compound (4.45–16.36 mg/g) also in PP, K2P or even KP. The samples PP, BP and K2P, but partially also RP, K1P and K3P contained notable peaks of glycerides of phenolic acids that could be attributed to *P. tremula*: e.g., *p*-coumaroyl glycerol, acetyl-*p*-coumraoylglycerol, 2-acetyl-1,3-di-*p*-coumaroylglycerol, 2-acetyl-3-*p*-coumaroyl-1-feruloylglycerol [79]. However, various glycerides of phenolic acids were found also in other natural sources such as aerial parts of *Asparagus officinalis* L. [81] or *Aegilops ovata* L. [82].

The samples KP, BP and K2P contained relevant amounts of terpene compounds. Among them KP and BP contained relevant abundance of abietic acid derivatives that could be attributed to *Pinus* resin but also other conifers [83]. Some small amounts of these compounds were present also in some other samples. BP was most rich in these compounds and also contained 6″-*O*-*p*-coumaroyltrifolin (kaempferol 3-(6-*p*-coumaroylgalactoside)), that was previously found in *Pinus sylvestris* L. needles [83]. In addition to this, in BP particular flavonols, e.g., myricetin-3,7,4′,5′-tetramethyl-ether, 3,7,4′-trimethylquercetin (ayanin) were found along with terpenes 18-acetoxy-*cis*-clerodan-3-ene-15-oic acid, 18-hydroxy-*cis*-clerodan-3-ene-15-oic acid, 15-hydroxy-*cis*-clerodan-3-ene-18-oic acid, 8-hydroxylabdan-15-oic acid, that were previously found in *Cistus* exudates [84].

Some samples (KP, PP, K2P, K3P and partially BP) contained also few peaks with pseudomoecular ion mass 453.3372 [M − H^+^]^−^, that could be tentatively identified as oleanoic acid, moronic acid or masticadienonic acid and could be potentially attributed as deriving from *Pistacia lentiscus* L. resin [85]. Samples KP and BP (traces) contained also small amounts of cupressic and isocupressic acid [75], that could be linked to *Cupressus* spp. [86] however totarol or agathadiol (considered markers of this type of propolis) were not detected [5]. This may suggest other conifers as possible sources of this propolis. Nevertheless, literature data showed also variability of totarol concentrations in different samples obtained from *Cupressus* spp. [86].

Considering all the data, notable contribution of *Poplar* species as source of propolis from Rab, Pag, Krk is found in contrast to the samples from Korčula and Biševo, which is consistent to the geographical location of the islands and distribution of poplars in Croatia [36]. Among the samples, RP contained particularly high levels of compounds typical for black poplar bud exudate, which may indicate dominating contribution of poplar in RP. In other samples its amount was at least several-fold lower, suggesting secondary contribution in total mass of propolis. The samples RP, K1P and K3P contained higher contribution of isoferulic acid than ferulic acid, which indicated *italica* variety of *P. nigra* as source plant. On the other side, the occurrence of various glycerides along with sakuranetin present in all samples (except BP) may be connected to other *Populus* species, such as *P. tremula*. These sources are quite common for European propolis from temperate climate zone [79]. On the other hand, some of the samples contained different terpenoids and unusual flavonoids that could be attributed to Mediterranean plants. Abietic and pimaric acid isomers as well as cupressic and isocupressic acids may be attributed to different, common coniferous tree species such as *Pinus, Cypressus* and *Juniperus*. These sources were also indicated as possible for other Mediterranean propolis, containing diterpenes, from Croatia but also west Algeria and Crete [15,87,88]. These compounds were more abundant in BP and KP. Some of the samples contained other compounds, possible to link with *Pistacia*. This source was suggested as one of the possible for Moroccan propolis [89]. The most interesting propolis, collected on island Biševo, distant from the land, was much different from other samples and contained compounds that could be linked to *Cistus* spp. Such origin was suggested also for diterpene propolis from Algeria which was very similar to the investigated Croatian sample [75]. Similarly, Tunisian propolis containing myricetin 3,7,4′,5′-methylether, that is typical for *Cistus* spp. leaf exudates, was recognized as source plant [90]. This confirms, that in areas where poplars are not always available, other plant sources can be used to form propolis.

### 3.4. Total Phenol Content and Antioxidant Potential

The total content of phenolic compounds assessed using Folin-Ciocalteu reagent ranged from 14.0 to 189.7 mg GAE/g of propolis for the investigated samples. The highest value was observed for RP and the lowest for BP, however all other values did not exceed 37 mg GAE/g. The total flavonoid content ranged between 7.2 and 103.9 mg QE/g of propolis, and was the highest in RP and lowest in K2P, but it did not exceed 18 mg QE/g for any other samples. These result are consistent with those obtained by UHPLC, where content of phenolics in RP was much higher than in other samples (Table 4). The values obtained for RP, were very similar to those reported for Chinese poplar propolis (233.98 mg GAE/g, 124.92 mg QE/g) and extracts from poplar buds (145.54 mg GAE/g, 126.23 mg QE/g) [123]. These values are consistent also with other obtained from other samples of Chinese poplar-type propolis that ranged from 87.11 to 257.93 mg GAE/g and 105.25 to 351.25 mg QE/g as well as those obtained for Croatian propolis 70–220 mg GAE/g [16,96]. This may suggest, that this sample was mostly originating form *Populus* exudates, while the other samples may contain no more than just a small percentage of this balsam. Similar observation was done for Anatolian propolis, where 3 different types were identified including those deriving from *P. nigra*, *P. tremula* and non-poplar type propolis. The amount of phenolics and flavonoids in the latter two, ranged from 11.24 to 47.15 mg GAE/g and from 3.88 to 48.70 mg QE/g, respectively [124]. Interestingly, the non-poplar type propolis from Anatolia was found to contain mainly Pinaceae and *Cistus* spp. pollen which suggest such plants to be major sources of these samples [124].

The antioxidant potential determined by DPPH ranged 2.6–81.6 mg GAE/g and in FRAP assay 0.1–0.8 mmol Fe^2+^/g. The highest activity was observed for RP and the lowest in BP. The antioxidant potential and phenolic/flavonoid content were positively (Table 5), significantly correlated (TP-DPPH *R*^2^ = 0.9368, TP-FRAP *R*^2^ = 0.7870, TF-DPPH *R*^2^ = 0.9019, TF-FRAP *R*^2^ = 0.7060, at *p* < 0.05) which links the activity with these groups of compounds. More varied activities in FRAP test ranging from 0.04 to 1.3 mmol Fe^2+^/g were found for Croatian propolis by Tlak-Gajger et al. [16]. 

Comparison of the obtained results with other reports on Croatian propolis was not possible, due to the different extraction, methodology or way of data presentation [18,78,125,126]. 

## 4. Conclusions

Typical propolis from Croatian islands along Adriatic Sea coast (Krk, Rab, Pag, Biševo and Korčula) were collected. The volatiles of the samples were isolated by HS-SPME and HD followed by GC-MS. The variability of the volatiles enabled differentiation of the samples in 2 groups of Mediterranean propolis: non-poplar type (dominated by α-pinene) and poplar type (cadinane type sesquiterpenes). Spectral variations (FT-MIR) associated with phenolics and other balsam-related components were significant among the samples. The quantitative data obtained from colorimetric tests and UHPLC-DAD suggests that only one sample was a typical black poplar-type propolis (characterized e.g., by abundance of caffeic acid prenyl esters, pinobanksin-3-*O*-acetate, pinocembrin). Few samples contained just its small, but visible contribution and derive mostly from other botanical sources such as other poplars or coniferous trees (e.g., *Pinus, Cupressus* or *Juniperus*). The latter may be linked with presence of abietic, dehydroabietic or pimaric acids, 6″-*O*-*p*-coumaroyltrifolin. One sample from Biševo was most particular and could be classified as Mediterranean diterpene propolis that derived *i.a.* from *Cistus* spp. exudates (characteristic compounds included myricetin-3,7,4′,5′-tetramethyl-ether, 15-hydroxy-*cis*-clerodan-3-ene-18-oic acid, 18-hydroxy-*cis*-clerodan-3-ene-15-oic acid, 18-acetoxy-*cis*-clerodan-3-ene-15-oic acid). The highest activity was observed for the samples of *Populus* origin. The antioxidant potential and phenolic/flavonoid content was positively, significantly correlated.

## Figures and Tables

**Table 1:** Volatiles determined by headspace solid-phase microextraction (HS-SPME)/gas chromatography (GC-MS).

No.	Compound	RI	BP			KP			RP			PP			K1P			K2P			K3P		
			I	II	III	I	II	III	I	II	III	I	II	III	I	II	III	I	II	III	I	II	III
1	Ethanol	< 900	0.2	0.1	-	-	-	-	-	-	-	-	0.2	-	-	-	-	1.0	0.6	-	0.6	0.8	-
2	Acetone	< 900	6.8	5.7	0.9	1.9	2.6	1.6	-	0.3	0.1	-	0.7	-	-	-	-	-	1.2	-	1.3	2.6	-
3	Isoprene	< 900	-	-	-	-	-	-	0.2	-	-	0.5	-	-	0.4	-	-	-	-	-	-	-	-
4	But-2-enal	< 900	-	-	-	0.3	-	-	-	-	-	-	-	-	-	-	-	-	-	-	-	-	-
5	Acetic acid	< 900	1.0	0.4	-	-	22.7	4.1	-	17.1	1.7	0.4	20.9	2.4	-	31.9	9.7	17.8	6.6	0.6	23.1	18.8	2.6
6	Propanoic acid	< 900	-	-	-	-	-	-	0.6	-	0.1	0.1	-	-	-	-	-	-	-	-	-	-	-
7	Butan-2-one	< 900	0.5	0.9	-	-	-	-	-	-	-	-	-	-	-	-	-	-	-	-	-	-	-
8	Pentanal	< 900	-	-	-	1.6	1.5	1.4	-	-	-	-	-	-	-	-	-	0.5	0.1	-	0.6	2.6	1.0
9	Heptane	< 900	0.2	0.1	-	-	-	-	-	-	-	-	-	-	-	-	-	-	-	-	-	-	-
10	2,5-Dimethylfuran	< 900	0.3	-	-	-	-	-	-	-	-	-	-	-	-	-	-	-	-	-	-	-	-
11	3-Methylbut-3-en-1-ol	< 900	-	-	-	-	-	-	0.1	2.1	0.6	1.7	1.2	0.5	1.7	0.8	0.7	-	-	-	1.7	1.8	1.7
12	2-Methylbut-2-enal	< 900	-	-	-	-	-	-	0.6	-	-	3.2	1.4	0.3	0.9	-	-	-	-	-	1.0	1.2	-
13	2-Methylpropanoic acid	< 900	-	-	-	-	-	-	-	1.5	0.5	-	-	-	-	0.6	-	-	-	-	-	-	-
14	3-Methylbut-2-en-1-ol	< 900	-	-	-	-	-	-	1.5	2.7	0.7	2.6	1.4	0.8	3.7	1.8	1.5	-	-	-	2.9	2.3	1.5
15	Toluene	< 900	2.1	1.0	0.3	0.3	-	-	-	-	-	-	-	-	-	-	-	-	-	-	-	-	-
16	3-Methylbut-2-enal	< 900	0.2	-	-	0.9	-	-	1.2	1.2	0.2	7.9	2.8	0.5	2.9	-	0.9	0.8	-	-	4.5	2.2	1.1
17	Oct-1-ene	< 900	0.3	0.3	-	-	-	-	-	-	-	-	-	-	-	-	-	-	-	-	-	-	-
18	Octane	< 900	0.4	0.3	-	-	-	-	-	-	-	0.2	-	-	-	-	-	-	-	-	-	-	-
19	Hexanal	< 900	-	-	-	1.9	1.3	1.0	-	-	-	-	-	-	-	0.6	0.6	1.7	0.7	0.2	1.2	1.8	0.7
20	2-Furancarboxaldehyde	< 900	0.2	-	-	0.9	0.3	-	-	-	-	-	-	-	-	-	-	1.0	-	-	0.8	-	-
21	2-Methylbutanoic acid	< 900	-	-	-	-	-	-	0.4	0.3	1.2	1.2	1.9	1.2	2.0	2.0	2.2	-	-	-	1.0	1.4	0.7
22	Non-1-ene	< 900	0.2	0.1	-	-	-	-	-	-	-	-	-	-	-	-	-	-	-	-	-	-	-
23	Styrene	< 900	0.9	0.1	-	-	-	-	0.4	0.3	0.1	0.6	-	-	2.7	0.8	0.6	-	-	-	0.8	0.3	0.6
24	Nonane	900	0.5	0.3	-	-	-	-	-	-	-	-	-	-	-	-	-	-	-	-	-	-	-
25	Heptanal	905	0.3	-	-	1.6	1.0	0.9	-	-	-	-	-	-	-	0.6	0.6	1.3	0.5	0.1	0.6	1.3	1.0
26	Prenyl acetate	927	-	-	-	-	-	-	0.9	2.4	0.6	0.9	0.7	0.3	2.6	1.0	1.0	-	-	-	-	-	-
27	Tricyclene	932	0.2	0.3	0.2	-	-	-	-	-	-	-	-	-	-	-	-	-	-	-	1.0	-	-
28	2-Methylbut-2-enoic acid	942	-	-	-	-	-	-	5.7	2.1	1.2	3.7	1.9	0.9	2.9	1.1	0.7	-	-	-	1.2	0.8	-
29	α-Pinene	943	32.9	42.2	52.7	8.8	7.6	13.5	-	-	0.6	5.1	5.9	3.6	1.3	1.0	1.3	-	-	-	-	1.2	1.7
30	Camphene	958	0.4	0.6	0.8	-	0.1	0.2	-	0.3	-	-	-	-	-	-	-	-	-	-	-	-	-
31	Verbenene	963	1.0	1.1	1.0	-	0.5	-	-	-	-	0.2	0.5	0.3	-	-	-	-	-	-	-	-	-
32	Benzaldehyde	970	0.4	-	-	2.7	0.7	0.5	2.1	0.3	0.2	5.2	0.7	0.4	9.8	2.0	1.9	17.9	5.7	3.2	4.7	2.6	3.2
33	β-Pinene	984	0.5	0.6	0.8	0.1	0.2	0.5	-	-	-	-	0.2	-	-	-	-	-	-	-	-	0.6	0.4
34	6-Methylhept-5-en-2-one	991	-	-	-	0.3	0.3	0.5	-	-	-	-	-	-	1.4	2.0	2.5	1.2	0.7	0.8	0.7	2.1	1.8
35	β-Myrcene	995	0.3	-	-	-	-	-	-	-	-	0.2	0.2	0.1	-	-	-	-	-	-	-	0.8	-
36	Octanal	1006	1.3	0.8	0.5	3.2	1.6	1.5	-	-	-	0.2	0.2	0.1	0.9	1.1	0.9	4.0	1.7	1.4	0.8	2.2	1.7
37	*p*-Mentha-1,5,8-triene	1010	0.4	0.3	-	-	-	-	-	-	-	-	-	-	-	-	-	-	-	-	-	-	-
38	δ-3-Carene	1016	0.6	0.5	0.6	0.7	0.5	0.8	-	-	-	0.5	0.2	0.1	-	-	-	-	-	-	-	-	-
39	o-Allyltoluene^*^	1019	0.2	0.1	-	-	-	-	-	-	-	-	-	-	-	-	-	-	-	-	-	-	-
40	α-Terpinene	1023	-	-	-	-	-	-	-	0.3	-	0.7	0.5	0.1	-	-	-	-	-	-	-	-	-
41	*p*-Cymene	1031	0.7	0.6	0.6	0.6	0.2	0.3	0.2	0.6	0.1	1.8	0.7	0.3	0.3	-	-	1.2	0.7	0.5	0.8	0.9	0.8
42	Limonene	1036	1.7	1.3	1.2	1.8	0.8	1.0	0.1	0.3	0.1	2.1	0.9	0.3	0.7	-	0.9	11.8	11.1	5.7	4.9	8.6	7.8
43	Benzyl alcohol	1046	0.5	0.1	0.1	0.5	0.2	0.2	6.9	2.7	2.1	1.5	0.5	0.6	3.7	1.7	2.0	-	-	-	3.1	3.6	5.3
44	Salicylaldehyde	1051	-	-	-	-	-	-	-	-	-	0.7	0.2	0.4	-	-	-	-	-	-	-	-	-
45	γ-Terpinene	1065	-	-	-	-	-	-	0.1	0.3	0.1	1.0	0.7	0.3	-	-	-	-	-	-	0.5	0.9	1.1
46	Acetophenone	1073	-	-	-	-	-	-	0.2	-	-	-	-	-	0.9	0.3	0.7	-	-	-	-	-	-
47	*p*-Cymenene	1094	2.4	1.0	0.9	0.6	0.6	0.7	0.1	0.3	-	0.9	0.5	0.4	-	-	-	-	-	-	-	-	-
48	Linalool	1103	-	-	-	-	-	-	0.3	1.2	0.4	1.0	0.9	0.6	3.0	1.8	1.9	0.3	0.3	0.3	1.6	1.3	0.7
49	Nonanal	1107	5.1	2.9	2.2	16.9	7.6	7.2	0.1	-	-	0.5	0.5	0.4	3.2	3.3	3.6	11.0	6.1	7.5	1.9	5.7	5.8
50	6-Methylhepta-3,5-dien-2-one	1110	-	-	-	6.8	3.8	3.5	-	-	-	-	-	-	5.3	4.2	5.5	-	-	0.5	0.8	1.7	1.5
51	2-Phenylethanol	1121	-	-	-	-	-	-	6.7	3.6	2.8	6.1	2.1	3.3	12.2	5.9	5.8	0.1	0.3	0.8	0.6	3.5	3.5
52	α-Campholenal	1132	1.6	1.4	1.3	1.0	0.7	0.7	-	-	-	-	-	-	-	-	-	-	-	-	-	-	-
56	*trans*-Pinocarveol	1147	0.6	1.4	1.2	0.1	0.5	0.6	-	-	-	-	-	0.3	-	-	-	-	-	-	-	-	-
54	*cis*-Verbenol	1149	0.2	0.5	0.5	-	-	-	-	-	-	-	-	-	-	-	-	-	-	-	-	-	-
55	*trans*-Verbenol	1153	2.2	4.1	4.8	0.6	1.6	2.0	-	-	-	-	0.5	0.3	-	-	-	-	-	-	-	-	-
56	Benzoic acid	1162	-	-	-	-	-	-	14.5	4.2	13.2	0.2	-	-	-	-	-	13.0	39.9	29.2	-	-	-
57	Benzyl acetate	1170	-	-	-	-	-	-	2.5	1.2	1.3	-	-	0.3	1.0	0.8	1.3	0.3	0.4	0.5	-	-	-
58	*trans-p*-Menth-2-ene-1,8-diol	1174	0.5	0.6	0.6	-	-	-	-	-	-	-	0.2	0.3	-	-	-	-	-	-	-	-	-
59	Terpinen-4-ol	1183	0.2	0.3	0.1	0.9	0.9	0.9	-	-	0.1	0.2	0.7	0.4	-	-	-	-	-	-	-	-	-
60	*p*-Cymene-8-ol	1191	0.8	0.9	1.5	0.1	0.6	1.0	-	-	-	0.2	0.5	0.5	-	-	-	-	-	-	-	-	-
61	α-Terpineol	1195	1.3	1.4	1.2	0.2	0.6	0.7	-	-	-	0.1	0.5	0.4	-	-	-	-	-	-	-	-	-
62	Myrtenal	1199	0.4	-	0.6	-	-	-	-	-	-	-	-	-	-	-	-	-	-	-	-	-	-
63	Myrtenol	1201	0.4	0.3	0.6	0.5	0.6	0.7	-	0.3	-	0.4	0.2	0.3	-	-	-	-	-	-	-	-	-
64	Decanal	1208	7.5	4.2	4.0	7.8	6.6	6.2	-	-	-	-	-	0.4	2.2	3.7	5.5	5.9	4.1	7.9	0.9	3.0	3.3
65	Verbenone	1213	2.3	3.2	3.1	0.1	0.6	0.6	-	-	-	-	-	0.1	-	-	-	-	-	-	-	-	-
66	β-Cyclocitral	1225	-	-	-	-	-	-	0.4	0.9	0.5	-	-	-	-	-	0.3	-	-	-	-	-	-
67	*trans*-Carveol	1226	2.3	1.8	1.5	0.2	0.1	0.5	-	-	-	-	-	-	-	-	-	-	-	-	-	-	-
68	2-Methoxy-*p*-cymene (Carvacrol methyl ether)	1249	-	-	-	8.5	6.0	6.9	-	-	-	-	0.2	-	-	-	-	-	-	-	-	-	-
69	Carvone	1250	0.5	0.4	-	-	-	-	-	-	-	-	-	-	-	-	-	-	-	-	-	-	-
70	Phenethyl acetate	1262	-	-	-	-	-	-	1.0	0.6	1.2	0.1	0.5	0.9	1.7	1.4	2.8	-	-	-	-	-	-
71	3-Phenylprop-2-enal	1276	-	-	-	-	-	-	0.1	-	0.2	-	-	-	-	-	0.1	-	-	-	-	-	-
72	Bornyl acetate	1289	0.3	0.4	0.5	1.1	2.4	3.1	-	-	-	-	0.2	-	-	-	-	-	-	-	-	-	-
73	Thymol	1301	1.6	1.1	1.4	-	-	0.1	-	-	-	-	-	-	0.3	1.0	1.6	4.5	5.5	18.9	15.8	10.1	39.9
74	*trans*-Cinnamyl alcohol	1315	-	-	-	-	-	-	-	-	0.5	-	-	-	-	-	-	-	-	-	-	-	-
75	α-Longipinene	1354	-	-	-	2.8	2.6	2.4	-	-	-	1.3	2.3	2.0	1.4	2.3	2.3	-	-	-	-	0.3	-
76	α-Cubebene	1355	0.9	1.0	-	-	-	-	-	-	-	-	-	-	-	-	-	-	-	-	-	-	-
77	Longicyclene	1374	-	-	-	2.6	2.6	2.9	-	-	-	-	-	-	2.0	3.9	6.1	-	-	-	-	-	-
78	α-Ylangene	1375	-	-	-	0.3	-	-	0.3	0.6	0.6	-	-	-	-	-	-	-	-	-	-	-	-
79	α-Copaene	1378	0.9	0.6	0.6	0.3	0.5	0.1	2.8	3.3	1.9	-	-	-	-	0.5	0.6	0.4	0.5	2.3	-	-	-
80	β-Bourbonene	1387	1.2	1.0	0.7	-	-	-	-	-	-	-	-	-	-	-	-	-	-	-	-	-	-
81	Geranyl acetate	1387	-	-	-	0.6	1.0	1.4	-	-	-	-	-	-	-	-	-	-	-	-	-	-	-
82	Tetradecane	1400	-	-	-	0.7	0.5	0.9	0.2	0.3	0.2	0.2	0.2	0.8	-	-	-	0.4	0.3	0.4	0.1	0.5	0.4
83	*cis*-Caryophyllene	1408	0.2	0.1	-	-	-	-	-	-	-	-	-	-	-	-	-	-	-	-	-	-	-
84	Junipene	1405	-	-	-	0.7	0.9	0.8	-	-	-	-	-	-	-	0.6	0.6	-	-	-	-	-	-
85	Vanillin	1407	-	-	-	-	-	-	-	-	-	-	-	-	-	-	-	1.9	8.1	10.7	-	-	-
86	Dodecanal	1411	0.4	0.1	0.2	0.1	0.3	0.5	-	-	-	-	-	-	-	-	-	-	-	-	-	-	-
87	*trans*-β-Caryophyllene	1422	2.7	2.3	2.6	2.3	2.4	2.9	-	0.9	-	-	-	-	-	-	-	-	-	-	-	-	-
88	α-Humulene	1456	0.5	0.5	0.5	0.2	0.5	0.5	0.1	0.3	0.1	-	-	-	-	-	-	-	-	0.1	-	-	-
89	Aromadendrene	1463	-	-	-	-	-	-	2.0	0.3	2.2	-	-	-	-	0.4	0.6	-	-	-	-	-	-
90	α-Amorphene	1479	-	0.1	-	-	-	-	2.3	2.7	3.5	-	-	-	-	0.4	0.7	-	-	-	-	-	-
91	Ar-curcumene	1485	-	-	-	-	-	-	0.9	0.9	1.2	-	-	-	-	-	-	-	-	-	-	-	-
92	α-Muurolene	1502	2.0	1.3	1.4	0.1	0.8	0.6	4.4	4.5	5.9	-	-	-	-	1.0	1.5	-	-	-	-	-	-
93	γ-Cadinene	1517	-	-	-	-	-	-	6.8	6.6	9.3	-	-	-	-	1.1	1.6	-	0.4	0.4	-	-	-
94	*cis*-Calamenene	1525	1.1	0.9	0.6	0.3	0.5	0.2	-	-	-	-	0.5	-	-	-	-	-	-	-	-	-	-
95	δ-Cadinene	1526	-	-	-	-	-	-	16.4	15.0	21.5	1.1	0.7	1.0	1.9	2.0	3.2	-	1.2	1.1	-	-	-
96	α-Cadinene	1540	-	-	-	-	-	-	1.4	1.2	2.1	1.3	1.9	3.3	0.1	1.7	2.0	-	-	-	-	-	-
97	α-Calacorene	1546	-	-	-	-	-	-	1.5	1.2	1.1	-	-	0.3	-	-	-	-	-	-	-	-	-
98	Caryophyllene oxide	1585	1.3	1.9	1.7	1.2	2.6	3.7	-	-	-	-	-	-	-	-	-	-	-	-	-	-	-
99	Guaiol	1601	-	-	-	-	-	-	1.8	0.9	2.7	18.5	14.3	28.9	8.9	3.2	6.5	-	-	-	2.3	2.6	2.9
100	Cedrol	1603	-	-	-	7.7	4.7	1.0	-	-	0.5	-	-	-	-	-	-	-	-	-	-	-	-
101	γ-Eudesmol	1636	-	-	-	-	-	-	-	-	-	1.7	1.6	3.7	2.0	1.1	1.7	-	-	-	-	-	-
102	α-Cadinol	1646	-	-	-	-	-	-	2.6	1.5	5.1	0.1	1.6	0.3	-	-	-	-	-	-	-	-	-
103	β-Eudesmol	1654	-	-	-	-	-	-	2.2	0.6	3.7	3.5	3.1	7.3	3.6	1.9	2.8	-	-	-	-	-	-
104	α-Eudesmol	1657	-	-	-	-	-	-	2.1	0.6	3.3	2.2	1.9	4.7	2.9	1.3	1.9	-	-	-	-	-	-
105	τ-Muurolol	1659	-	-	-	-	-	-	1.2	0.6	1.7	-	-	-	-	-	-	-	-	-	-	-	-
106	Bulnesol	1672	-	-	-	-	-	-	0.6	-	-	10.7	7.7	16.7	-	-	-	-	-	-	-	-	-
107	Benzyl benzoate	1767	-	-	-	-	-	-	-	-	-	-	-	-	-	-	-	-	1.0	0.8	-	-	-
108	Hexadecanal	1818	-	-	-	0.2	0.8	1.0	-	-	-	-	-	-	-	-	1.9	-	-	-	-	-	-

BP, KP, RP, PP, K1P, K2P, K3P—the codes of the samples (Section 2.1.); I—Carboxen (CAR)/Polydimethylsiloxane (PDMS) fiber; II—Divinylbenzene (DVB)/CAR/PDMS fiber, III—PDMS/DVB fiber, RI = retention indices on HP-5MS column; - = not identified, *—correct isomer not identified.

**Table 2:** Volatiles obtained by hydrodistillation (HD/GC-MS).

No.	Compound	RI	BP	KP	RP	PP	K1P	K2P	K3P
1	1,3-Dimethylbenzene	< 900	-	-	-	-	-	-	0.1
2	Ethenylbenzene	< 900	-	-	-	-	-	-	0.1
3	Nonane	900	0.2	0.1	-	-	-	-	0.1
4	α-Pinene	942	11.3	0.2	-	0.1	-	-	0.1
5	Camphene	958	0.2	-	-	-	-	-	-
6	Verbenene	962	0.3	-	-	-	-	-	-
7	Benzaldehyde	969	-	-	0.1	-	-	-	0.1
8	β-Pinene	984	0.2	-	-	-	-	-	-
9	Octanal	1005	0.2	0.1	-	-	0.1	0.1	0.2
10	δ-3-Carene	1015	0.2	-	-	-	-	-	-
11	*p*-Cymene	1031	0.2	-	-	-	-	-	-
12	Limonene	1035	0.2	-	-	-	-	-	0.1
13	Nonanal	1106	0.5	0.3	-	-	0.1	0.2	0.3
14	α-Campholene aldehyde	1132	0.5	-	-	-	-	-	-
15	*cis*-Verbenol	1149	1.0	-	-	-	-	-	-
16	*trans*-Verbenol	1153	2.1	-	-	-	-	-	-
17	*cis*-*p*-Menth-2-ene-1,8-diol	1156	0.8	-	-	-	-	-	-
18	Pinocarvone	1167	0.2	-	-	-	-	-	-
19	Benzyl acetate	1170	-	-	0.1	-	-	-	-
20	*trans*-*p*-Menth-2-ene-1,8-diol	1174	2.9	-	-	-	-	-	-
21	Terpinen-4-ol	1183	0.2	-	-	-	-	-	-
22	Octanoic acid	1187	-	-	0.1	-	0.1	-	0.1
23	4-Methylacetophenone	1189	0.2	-	-	-	-	-	-
24	*p*-Cymene-8-ol	1191	0.6	-	-	-	-	-	-
25	α-Terpineol	1195	0.6	-	-	-	-	-	-
26	Myrtenal	1199	0.3	-	-	-	-	-	-
27	Myrtenol	1201	0.6	-	-	-	-	-	-
28	Decanal	1208	1.5	0.9	0.1	0.2	0.7	1.1	0.8
29	Verbenone	1213	2.3	-	-	-	-	-	-
30	β-Cyclocitral	1225	-	-	0.1	-	-	-	-
31	*trans*-Carveol	1226	1.0	-	-	-	-	-	-
32	3-Phenylbutan-2-one	1249	-	-	-	-	0.1	-	0.1
33	4-Methoxybenzaldehyde	1260	-	-	-	-	-	-	0.1
34	Phenethyl acetate	1262	-	-	0.2	-	-	-	-
35	3-Phenylprop-2-enal	1276	-	-	-	-	0.2	-	0.2
36	Nonanoic acid	1284	0.2	0.2	0.1	0.1	0.4	0.1	0.1
37	Bornyl acetate	1289	0.2	-	-	-	-	-	-
38	Thymol	1301	0.8	-	-	-	0.2	1.3	2.4
39	Carvacrol	1312	0.2	-	-	-	-	-	-
40	2-Methoxy-4-vinylphenol	1319	-	-	0.2	0.1	0.2	7.3	0.6
41	α-Longipinene	1354	-	0.1	-	-	0.1	-	-
42	4-Phenylbut-3-en-2-one^*^	1362	-	-	0.1	0.1	0.2	-	0.1
43	Eugenol	1363	-	-	-	0.2	-	0.1	-
44	4-Ethenyl-1,2-dimethoxybenzene	1373	-	-	0.2	-	0.6	-	1.2
45	Longicyclene	1374	-	0.1	-	-	-	-	-
46	α-Copaene	1378	0.2	-	0.2	-	-	-	-
47	Decanoic acid	1381	0.3	0.2	0.1	0.1	0.2	0.2	0.1
48	β-Bourbonene	1387	0.2	-	-	-	-	-	-
49	Tetradecane	1400	-	0.1	-	-	0.1	0.1	0.1
50	Junipene	1405	-	-	-	-	-	-	0.1
51	Dodecanal	1411	0.3	0.1	-	-	0.1	0.2	0.1
52	*trans*-β-Caryophyllene	1422	0.8	0.1	0.1	-	-	-	-
53	α-Humulene	1456	0.2	-	-	-	-	-	-
54	Aromadendrene	1463	-	-	0.6	-	-	0.1	-
55	α-Amorphene	1479	0.2	-	0.8	-	0.1	-	-
56	Pentadecane	1500	-	-	-	-	-	-	0.1
57	α-Muurolene	1502	1.0	0.1	2.0	-	0.2	0.2	-
58	γ-Cadinene	1517	0.3	-	3.1	-	0.2	0.3	-
59	δ-Cadinene	1526	0.8	0.3	6.6	0.1	0.4	0.7	0.6
60	α-Cadinene	1540	-	-	1.4	-	0.9	-	-
61	α-Copaen-11-ol	1541	-	-	-	0.9	-	-	0.6
62	α-Calacorene	1546	0.2	-	1.2	-	0.1	-	-
63	Dodecanoic acid	1578	0.2	-	-	-	0.5	0.6	-
64	Caryophyllene oxide	1585	5.8	0.5	-	-	-	0.1	-
65	Guaiol	1601	1.6	3.1	4.4	14.3	4.6	-	5.2
66	γ-Eudesmol	1636	-	-	5.5	3.9	2.6	0.3	1.6
67	α-Cadinol	1646	0.5	-	10.1	-	0.5	0.8	0.6
68	α-Muurolol (torreyol)	1652	0.5	-	1.9	-	-	0.3	-
69	β-Eudesmol	1654	-	-	9.6	6.4	5.1	0.5	2.7
70	α-Eudesmol	1657	-	-	9.4	4.5	4.0	-	1.9
71	τ-Muurolol	1659	-	-	-	-	-	1.4	-
72	Bulnesol	1672	-	-	2.5	15.9	2.1	-	2.4
73	Heptadecane	1700	0.2	-	0.1	0.5	-	0.1	-
74	Benzyl benzoate	1767	0.2	-	0.1	-	0.1	13.8	0.1
75	Tetradecanoic acid	1772	-	-	-	-	-	-	0.1
76	Hexadecanal	1818	1.0	2.9	-	-	3.8	1.6	3.3
77	Benzyl salycilate	1870	-	-	-	-	-	2.7	-
78	Nonadecane	1900	1.0	0.3	0.2	1.0	0.6	0.5	0.3
79	Heptadecan-2-one	1903	-	-	0.1	0.3	0.1	-	0.2
80	Hexadecanoic acid	1972	-	0.8	0.1	0.6	1.0	0.4	0.9
81	Manoyl oxide	1990	8.7	0.3	0.2	0.5	0.1	-	-
82	Eicosane	2000	2.9	0.6	0.1	0.1	0.2	0.2	0.1
83	Octadecan-2-one*	2004	-	-	0.1	0.5	0.2	-	0.5
84	Dehydroabietan	2054	3.6	-	-	-	-	-	-
85	Manool	2055	-	5.7	0.6	0.3	0.5	-	-
86	Octadecan-1-ol	2074	-	0.3	-	-	0.5	-	2.8
87	Abietadiene	2077	3.1	-	-	-	-	-	-
88	Benzyl cinnamate	2091	-	-	-	-	-	14.9	0.1
89	Heneicosane	2100	3.2	4.1	1.0	3.5	2.3	3.7	2.2
90	Nonadecan-2-one	2105	-	-	0.1	1.2	0.9	-	1.6
91	Docosane	2200	4.2	13.5	11.2	12.7	23.5	2.7	26.0
92	Methyl sandaracopimarate	2252	0.6	-	-	-	-	-	-
93	Dehydroabietal	2261	2.4	-	-	-	-	-	-
94	(*Z*)-Tricos-9-ene	2272	0.5	1.6	0.3	1.5	1.7	2.9	1.6
95	Methyl isopimarate	2290	1.1	-	-	-	-	-	-
96	Tricosane	2300	11.6	31.8	5.2	22.3	24.8	35.1	27.7
97	Abietadien-18-al	2301	2.6	-	-	-	-	-	-
98	Dehydroabietic acid	2350	1.0	-	-	-	-	-	-
99	Tetracosane	2400	-	25.4	9.4	-	4.3	-	-

BP, KP, RP, PP, K1P, K2P, K3P—the codes of the samples (Section 2.1.); RI = retention indices on HP-5MS column; - = not identified, *—correct isomer not identified.

**Table 3:** Compounds identified by ultra high performance liquid chromatography with diode array detector and quadrupole time-of-flight mass spectrometry (UHPLC-DAD-QqTOF-MS) in extracts of Croatian propolis samples.

No.	Component	RT	UV max [nm]	[M − H^+^]^−^	[M + H^+^]^+^ / [M + Na^+^]^+^/ [M − H_2_O + H^+^]^+^	BP	KP	RP	PP	K1P	K2P	K3P	References
1	4-Hydroxybenzoic acid ^a,b,c^	7.33	256	137.0246	139.0388	−	−	+	tr	+	+	+	[91]
2	3-Hydroxybenzoic acid ^b,c^	7.97	258	137.0247	139.0398	tr	−	−	tr	−	+	tr	[91]
3	4-Hydroxybenzaldehyde ^b,c^	9.46	282	121.0296	123.0440	−	−	−	−	−	+	−	[92]
4	Caffeic acid ^a,b,c^	11.02	323, 295sh	179.0351	181.0498	tr	tr	++	+	+	+	+	[79,91,93,94,95,96,97,98]
5	Vanillin ^a,b,c^	12.41	310,280, 230	151.0404	153.0543	tr	tr	tr	+	tr	++	tr	[86,91]
6	Benzoic acid ^a,b,c^	13.46	230, 274	121.0296	123.0434	tr	tr	+	tr	tr	++	tr	[86,91]
7	*p*-Coumaric acid ^a,b,c^	13.81	310, 300sh	163.0401	165.0542	+	tr	++	+	+	++	+	[79,86,91,94,95,96,97]
8	*p*-Coumaroyl glycerol ^b,c^	13.94	310, 300sh, 229	237.0773	−/261.0733	tr	−	tr	+	tr	+	tr	[60,99,100]
9	Ferulic acid ^a,b,c^	14.63	322, 298sh	193.0497	195.0641	+	tr	+	+	+	++	+	[79,86,91,94,95,97]
10	Isoferulic acid ^a,b,c^	14.74	324, 300sh	193.0497	195.0660	tr	−	++	+	++	+	++	[79,86,91,94,95,97]
11	*Caffeoylmalic acid (Phaseolic acid) isomer ^b,c^	15.29	328, 298sh	295.0824	−/319.0778	−	−	+	−	−	+	−	[101,102]
12	4-Hydroxy-3-methoxycinnamaldehyde (Coniferyl aldehyde) ^b,c^	15.37	339	177.0556	179.0701	tr	−	−	−	−	++	−	[103,104]
13	*p-Coumaric acid derivative^b^	15.64	310, 225	329.1042	−/353.0977	−	−	−	tr	−	+	−	−
14	*p-Coumaric acid derivative^b^	15.72	310, 226	359.1134	−/383.1081	tr	−	−	tr	−	+	tr	−
15	*p-Coumaric acid derivative^b^	15.83	310, 227	359.1137	−/383.1090	tr	−	−	+	−	++	tr	−
16	*p-Coumaric acid derivative^b^	15.90	311, 228	359.1137	/383.1087	tr	−	−	+	−	+	−	−
17	*Aromadendrin (dihydrokaempferol) ^b,c^	15.98	292	287.0559	289.0705	tr	++	+	+	+	+	+	[105]
18	*Ferulic acid derivative ^b^	16.09	322, 298sh	389.1253	−/413.1204	tr	−	−	+	−	+	tr	−
19	*Ferulic acid derivative ^b^	16.18	322, 298sh	389.1239	−/413.1200	+	−	−	+	−	++		−
20	*Acetyl-*p*-coumraoylglycerol ^b, c^	16.29	311	279.0879	−/303.0829	+	−	tr	+	tr	++	tr	[60,99,100]
21	Apigetrin (apigenin 7-*O*-glucoside) ^b, c^	16.30	315sh, 265	431.0976	−/455.0966	−	−	+	tr	−	−	−	[94,95]
22	Dimethylcaffeic acid (DMCA) ^b, c^	16.40	324, 294sh	207.0664	209.0943	−	tr	++	+	+	tr	+	[94,95]
23	Cinnamic acid ^a,b,c^	16.75	278	147.0444	149.0601	tr	−	+	tr	tr	+	tr	[6,91,94,95]
24	*Caffeic acid derivative ^b^	17.15	328, 298sh	277.1082	−/301.1052	−	−	+	−	+	−	tr	−
25	Pinobanksin 5-methylether ^b,c^	17.32	288	285.0762	287.0884	−	−	++	tr	tr	tr	+	[6,94,95,96,98]
26	Eriodyctiol (4′-hydroxynaringenin) ^b,c^	17.39	288	287.0562	289.0695	−	+	−	+	−	tr	+	[95]
27	Pinusenocarp ^b,c^	17.49	−	291.1597	293.1749	+	tr	−	tr	−	−	−	[106]
28	*Quercetin dimethyl ether ^b,c^	17.69	363, 245	329.0667	331.0809	tr	−	++	tr	tr	−	−	[91,94,96]
29	6″-*O*-*p*-Coumaroyltrifolin (Kaempferol 3-(6-*p*-coumaroylgalactoside) ^b,c^	17.71	350sh, 313, 262	593.1290	595.1483	+	−	−	−	−	−	−	[83]
30	Quercetin ^a,b,c^	17.89	364, 270sh, 265	301.0349	303.0488	+	+	++	+	tr	+	+	[6,91,94,95]
31	Luteolin ^a,b,c^	17.93	345, 254	285.0407	287.0553	+	−	+	tr	tr	tr	tr	[91,95]
32	*Caffeic acid derivative ^b^	18.10	328, 329sh	349.1658	−/373.1628	−	−	+	tr	tr	−	+	−
33	1-Caffeoyl-3-*p*-coumaroyl glycerol ^b,c^	18.37	315, 298sh, 235	399.1085	401.1190	−	−	−	−	−	+	−	[60,79,95]
34	Pinobanksin ^a,b,c^	18.45	292	271.0611	273.0763	tr	+	++	+	+	tr	+	[79,94,95,96]
35	Quercetin 3-methyl ether ^b,c^	18.46	355, 268sh, 255	315.0497	317.0657	+	+	+	+	+	+	+	[94]
36	7,4′-Di-*O*-methylmyricetin ^b,c^	18.60	361, 259	345.0608	347.0764	+	−	−	−	−	−	−	[107]
37	Caffeoyl-feruloylglycerol ^b,c^	18.64	326, 298sh, 240	429.1175	−/453.1153	+	−	+	+	+	+	−	[60,99]
38	Chrysin-5-methyl ether ^b,c^	18.70	314sh, 264	267.0663	269.0814	tr	tr	+	tr	tr	tr	tr	[98,108]
39	Hesperetin	18.69	290	301.0716	303.0851	−	+	+	+	−	−	−	[109]
40	Naringenin ^a,b,c^	18.92	289	271.0612	273.0746	tr	++	+	+	tr	+	+	[95,108]
41	*Caffeic acid derivative ^b^	19.17	321	299.0932	323.0905	−	−	+	+	+	−	+	−
42	Apigenin ^a,b,c^	19.26	338, 290sh, 263	269.0450	271.0592	tr	tr	++	tr	+	+	tr	[6,94,95]
43	Kaempferol ^a,b,c^	19.44	366, 295sh, 265	285.0403	287.0544	tr	+	+	+	+	+	+	[6,79,91,94,95]
44	*β*-Styrylacrilic acid (cinnamylideneacetic acid) ^b,c^	19.52	311, 240sh	173.0613	175.0757	−	−	++	−	tr	−	tr	[94,110]
45	1,3-Di-*p*-coumaroylglycerol ^b,c^	19.57	312, 300sh	383.1129	−/407.1096	+	−	−	+	+	+	+	[60,79]
46	Isorahmnetin (quercetin 3′-methyl ether) ^a,b,c^	19.72	371, 268sh, 256	315.0502	317.0661	+	+	+	+	+	+	+	[6,79,91,95,96]
47	*p*-Coumaroyl-feruloylglycerol ^b,c^	19.85	316, 298sh	413.124	−/437.1196	+	−	+	+	+	+	tr	[60,86]
48	2-Acetyl-1,3-di-caffeoylglycerol ^b,c^	19.92	328, 298sh	457.1133	−/481.1099	+	−	+	+	+	+	+	[95,97]
49	Caffeic acid butenoic or isobutenoic ester ^b,c^	19.98	326, 298sh, 245	233.0827	235.0969/ 257.0800	−	−	+	−	+	−	+	[60,111]
50	Luteolin-5-methyl ether ^b,c^	20.06	350, 298sh, 267	299.0549	−/323.0543	+	+	+	+	+	+	+	[94]
51	Di-1,3-feruloylglycerol ^b, c^	20.07	323, 298sh	443.1329	−/467.1300	+	−	−	−	−	++	−	[60,79,95]
52	*Quercetin-dimethyl ether ^b,c^	20.23	358, 260	329.066	331.0808	+	+	+	+	tr	−	+	[91,94,96]
53	Galangin-5-methyl ether ^b,c^	20.26	352, 300sh, 260	283.0602	285.0726	tr	+	+	+	tr	−	+	[94,96]
54	Quercetin-3,3′-dimethyl ether ^b,c^	20.36	356, 269sh, 255	329.0651	331.0809	+	tr	+	tr	tr	tr	+	[94,95]
55	Myricetin 3,7,4′-trimethyl ether ^b, c^	20.63	344, 266	359.0772	361.0922	+	−	−	−	−	−	−	[112]
56	*Hydroxy-tetramethoxyflavone ^c^	20.63	370, 282	357.0976	359.1118/ 381.0954	−	−	+	−	−	tr	tr	−
57	Caffeic acid prenyl or isoprenyl ester I ^b,c^	20.69	324, 298sh	247.0987	249.1634	tr	−	+	+	+	−	+	[91,94,95,96,98]
58	Caffeic acid butyl or isobutyl ester ^b, c^	20.73	326, 298sh	235.0972	−	−	tr	+	+	+	−	+	[79,113]
59	*p-Coumaric acid derivative ^b^	20.77	312, 282	445.1651	−/469.1612	−	−	−	−	−	+	−	−
60	Rhamnetin (quercetin 7-methyl ether) ^b,c^	20.91	356, 268sh, 256	315.0504	317.0639	+	+	+	+	+	+	+	[6,95,114]
61	Caffeic acid prenyl or isoprenyl ester II ^b,c^	21.04	325, 298sh	247.0979	249.1273	tr	tr	++	+	++	tr	++	[91,94,95,96,98]
62	2-Acetyl-1-caffeoyl-3-*p*-coumaroylglycerol ^b,c^	21.22	316, 299sh	441.1182	−/465.1147	+	−	−	+	−	+	−	[79,95,97,99]
63	Caffeic acid prenyl or isoprenyl ester III ^b,c^	21.23	324, 298sh	247.0976	−/271.1105	tr	tr	++	+	++	+	++	[91,94,95,96,98]
64	Caffeic acid prenyl or isoprenyl ester IV ^b,c^	21.33	325, 298sh	247.0973	249.1123/271.099	tr	tr	++	+	++	tr	++	[91,94,95,96,98]
65	*Quercetin dimethyl ether ^b,c^	21.43	368, 254	329.0659	331.0801	tr	+	tr	+	−	tr	+	[91,94,95,96,98]
66	2-Acetyl-1-caffeoyl-3-feruloylglycerol ^b,c^	21.50	322, 300sh	471.1300	495.1259	tr	−	−	tr	tr	+	tr	[97,99]
67	Caffeic acid benzyl ester ^b,c^	21.65	328, 298sh	269.0818	271.0971	tr	+	+	+	+	tr	+	[94,95,96]
68	Quercetin-3,7-dimethyl ether ^b,c^	21.66	356, 268sh, 256	329.0674	331.0827	+	tr	tr	tr	tr	tr	tr	[91,95]
69	*3,5,2′-Trihydroxy-7,8,4′-trimethoxyflavone ^b,c^	21.79	360, 256	359.0768	361.0927	+	tr	−	tr	tr	+	+	[115]
70	Chrysin ^a,b,c^	21.93	312sh, 268	253.0505	255.0659	tr	+	+	+	++	+	++	[79,91,94,95,96,98]
71	Pinocembrin ^a,b,c^	22.12	290	255.0666	257.0799	+	tr	++	+	+	tr	+	[91,94,95,96,98]
72	Caffeic acid phenethyl ester ^b,c^	22.36	325, 295	283.0984	285.0940	tr	−	++	tr	+	tr	tr	[91,94,95,96,98]
73	Sakuranetin ^b,c^	22.38	290	285.0773	287.0908	tr	+	+	++	+	++	++	[91,95,108]
74	Galangin ^a,b,c^	22.43	360, 266	269.0454	271.0761	tr	tr	+	tr	tr	tr	tr	[79,91,95,96,98]
75	*p-Coumaric derivative ^c^	22.52	311	325.109	−/349.1033	−	−	−	−	−	+	−	−
76	*Pinobanksin-7-methyl ether ^b, c^	22.62	290	285.0777	287.0898	−	tr	+	+	+	tr	+	[94]
77	2-Acetyl-1,3-di-*p*-coumaroylglycerol ^b,c^	22.72	312, 300	425.1232	−/449.1202	+	−	−	+	tr	++	tr	[79,95,99]
78	Pinobanksin 3-O-acetate ^b,c^	22.80	295	313.0713	315.0875	tr	tr	++	tr	+	tr	+	[79,94,95,96]
79	Kaempferide (kaempferol 4′-methyl ether) ^b,c^	22.93	365, 267	299.0555	301.0698	tr	++	+	+	tr	tr	+	[91]
80	*p*-Coumaric acid prenyl or isoprenyl ester I ^b,c^	23.11	311, 299sh	231.1028	233.1178	−	tr	+	+	+	−	+	[94,96,98]
81	2-Acetyl-3-*p*-coumaroyl-1-feruloylglycerol ^b,c^	23.12	318, 299sh	455.134	479.1310	+	−	−	+	+	++	+	[79,97]
82	Methoxychrysin ^b,c^	23.21	310sh, 266, 245sh	283.0611	285.0714	−	tr	+	tr	tr	−	+	[94,96,110]
83	*p*-Coumaric acid prenyl or isoprenyl ester II ^b,c^	23.38	310, 299sh	231.1025	−/255.1003	−	tr	+	+	+	+	tr	[94,96,98]
84	2-Acetyl-1,3-di-feruloylglycerol ^b,c^	23.62	328, 298sh	485.1423	487.1578	+	−	−	+	−	+	−	[97,100]
85	Kaempferol 3,4′-dimethyl ether ^b,c^	23.72	350, 267	313.0722	315.0854	+	++	−	+	−	tr	+	[116,117]
86	Cupressic acid ^b,c^	23.82	−	319.2287	321.2437	tr	+	−	−	−	−	−	[75]
87	*p*-Coumaric acid benzyl ester ^b,c^	23.88	312, 298sh	253.0870	−/277.0826	+	−	+	+	tr	++	+	[79,94,96]
88	*15-Hydroxy-*cis*-clerodan-3-ene-18-oic acid ^b,c^	23.95	−	321.2442	323.2589	+	−	−	−	−	−	−	[75]
89	18-Hydroxy-*cis*-clerodan-3-ene-15-oic acid ^b,c^	24.14	−	321.2449	−/305.2481	+	−	−	−	−	−	−	[75]
90	Isocupressic acid ^b,c^	24.20	−	319.2284	321.2445/ 303.2332	−	+	−	−	−	−	−	[75]
91	Caffeic acid cinnamyl ester ^b,c^	24.32	326, 300sh	295.0971	−/319.0945	−	−	++	tr	+	tr	+	[94,95,110]
92	Ferulic acid benzyl ester^* b,c^	24.65	326, 298	283.0968	285.0725	+	−	+	++	+	++	+	[79,86,111]
93	3,7,4′-Trimethylquercetin (ayanin) ^b,c^	24.78	355, 255	343.0825	345.0977	+	−	−	−	−	−	−	[90]
94	Pinobanksin 3-*O*-propanoate ^b,c^	25.05	294	327.0876	329.1023/ 351.0835	−	tr	++	tr	+	tr	+	[94,95,96]
95	*p*-Coumaric acid phenethyl ester ^b,c^	25.06	310, 300sh	267.1033	−/291.0991	−	tr	+	+	+	+	+	[98]
96	*Hydroxydehydroabietic acid isomer ^b,c^	25.00	−	315.1969	317.2118	+	tr	−	tr	tr	−	−	[106]
97	Myricetin-3,7,4′,5′-tetramethyl-ether ^b,c^	25.11	344, 265	373.0937	375.1088	++	−	−	−	−	−	−	[75,90,118,119,95]
98	*p*-Coumaric acid cinnamyl ester ^b,c^	26.83	312, 300sh	279.1024	−/303.0986	−	tr	+	tr	tr	tr	tr	[95]
99	*Trihydroxytriterpene carboxylic acid ^c^	26.41	−	487.3439	489.3595	−	++	−	++	−	++	++	−
100	*Hydroxyditerpene carboxylic acid ^b,c^	26.80	−	321.2439	−/345.2411	+	−	−	−	−	−	−	[120]
101	Pinobanksin 3-*O*-butanoate or isobutanoate ^b,c^	26.93	293	341.1022	343.1178	−	+	+	+	−	+	+	[94,96]
102	Pinostrobin chalcone ^b,c^	26.94	339, 287sh	269.0811	271.0972	tr	−	++	+	+	+	+	[95]
103	*Trihydroxyflavanone	27.03	267, 290	271.0977	273.1115	tr	−	++	+	+	−	+	−
104	Pinobanksin 3-*O*-pentenoate or isopentenoate I ^b,c^	27.06	295	353.1038	355.1181	−	−	++	−	tr	tr	+	[95,96,121]
105	Pinostrobin (pinocembrin-7-methyl ether) ^a,b,c^	27.20	289	269.2126	−/293.2093	−	tr	tr	+	tr	tr	+	[95]
106	*8-Hydroxylabdan-15-oic acid ^b,c^	27.26	−	323.2601	−/347.2569	++	−	−	−	−	−	−	[84]
107	Pinobanksin 3-*O*-pentanoate or isopentanoate II ^b,c^	27.68	292	355.1198	357.1342/ 379.1159	−	+	+	+	+	tr	+	[94,95,96]
108	Methoxycinnamic acid cinnamyl ester ^b,c^	27.74	280	293.2131	295.2278/ 317.2098	−	−	+	+	+	+	+	[94,110]
109	*18-Acetoxy-*cis*-clerodan-3-ene-15-oic acid ^b,c^	28.08	−	363.2544	−/387.2521	+	−	−	−	−	−	−	[84]
110	Dehydroabietic acid isomer ^b,c^	28.16	−	299.2023	301.2173	++	+	tr	+	+	+	tr	[122]
111	Pinobanksin 3-*O*-hexanoate ^b,c^	28.21	282	369.1349	371.1497	−	−	+	−	−	−	tr	[94,96,110,121]
112	Abietic or pimaric acid isomer I ^b,c^	28.59	−	301.2175	303.2327	+	+	−	tr	+	tr	−	[106]
113	Abietic or pimaric acid isomer II ^b,c^	28.70	−	301.2173	303.2328	+	+	−	tr	tr	−	−	[106]
114	Abietic or pimaric acid isomer III ^b,c^	28.83	−	301.2180	303.2326	++	+	−	tr	−	−	−	[106]
115	Abietic or pimaric acid isomer IV ^b,c^	29.06	−	301.2180	303.2318	+	−	−	−	+	−	−	[106]
116	*Oleanoic acid ^b,c^	29.39	−	453.3372	455.3549	+	++	−	++	−	++	+	[85]
117	*Moronic acid ^b,c^	29.60	−	453.3372	455.3549	−	+	−	+	−	+	+	[85]
118	*Masticadienonic acid ^b,c^	29.70	−	453.3372	455.3549	tr	+	−	+	−	+	+	[85]

* Component tentatively identified; ^a^ Confirmed with standard; ^b^ Confirmed with HR-MS, MS/MS (data not shown) and/or UV; ^c^ Confirmed with references; ++/+ compound detected (different relative abundance); − compound not detected; tr—compound found in traces.

**Table 4:** Quantities of the selected compounds identified by UHPLC-DAD-QqTOF-MS in extracts of Croatian propolis samples.

No.	Compound	Rt [min]							
			BP	KP	RP	PP	K1P	K2P	K3P
			[mg/g]						
1	Caffeic acid	11.02	tr	tr	6.80	0.15	0.68	0.22	0.51
2	Vanillin	12.41	tr	tr	tr	1.89	tr	8.71	tr
3	Benzoic acid	13.46	tr	tr	8.11	tr	tr	17.96	tr
4	*p*-Coumaric acid	13.81	0.17	tr	2.60	0.41	0.54	4.22	0.48
5	Ferulic acid	14.63	0.34	tr	1.78	0.54	0.34	4.10	0.31
6	Isoferulic acid	14.74	tr	nd	8.30	0.17	0.80	0.09	0.67
7	Dimethylcaffeic acid^a^	16.40	nd	tr	11.57	0.47	4.01	tr	2.59
8	Cinnamic acid	16.75	tr	nd	6.12	tr	tr	1.93	tr
9	Pinobanksin 5-methylether^b^	17.32	nd	nd	17.80	tr	tr	tr	0.83
10	Quercetin	17.89	0.38	0.88	1.82	0.66	tr	0.47	0.76
11	Pinobanksin	18.45	tr	0.20	13.53	0.21	0.16	tr	0.43
12	Chrysin-5-methyl ether^c^	18.70	tr	tr	0.19	tr	tr	tr	tr
13	Naringenin	18.92	tr	1.43	0.71	0.90	tr	0.46	0.73
14	Apigenin	19.26	tr	tr	3.80	tr	0.10	0.34	tr
15	Kaempferol	19.44	tr	1.40	3.01	1.64	0.13	1.76	1.72
16	Isorhamnetin	19.72	0.20	0.44	1.76	0.35	0.05	0.14	0.29
17	Luteolin-5-methyl ether^d^	20.06	0.24	0.32	12.28	2.91	1.74	0.54	1.54
18	Galangin 5-methyl ether^e^	20.26	tr	0.85	1.66	0.02	tr	nd	0.20
19	Quercetin 3,3′-dimethyl ether^f^	20.36	0.52	tr	1.26	tr	tr	tr	0.03
20	Myricetin 3,7,4′-trimethyl ether^f^	20.63	0.67	nd	nd	nd	nd	nd	nd
21	Rhamnetin (quercetin-7-methyl ether)	20.91	0.08	0.09	1.94	0.18	0.15	0.14	0.35
22	Caffeic acid prenyl or isoprenyl ester II^a^	21.04	tr	tr	14.07	0.28	3.36	tr	1.66
23	Caffeic acid prenyl or isoprenyl ester III^a^	21.23	tr	tr	20.05	0.73	6.19	1.56	3.06
24	Caffeic acid prenyl or isoprenyl ester IV^a^	21.33	tr	tr	3.62	0.15	1.94	nd	1.20
25	*Quercetin dimethyl ether^f^	21.43	tr	0.63	tr	0.53	nd	tr	0.67
26	Caffeic acid benzyl ester^a^	21.65	tr	0.05	12.11	0.16	0.85	tr	0.75
27	Quercetin-3,7-dimethyl ether^f^	21.66	0.15	tr	tr	tr	tr	tr	tr
28	Chrysin	21.93	tr	0.04	30.71	0.70	7.50	0.17	5.72
29	Pinocembrin	22.12	0.11	tr	39.86	0.81	3.50	tr	2.03
30	Caffeic acid phenethyl ester^a^	22.36	tr	nd	9.31	tr	4.69	tr	tr
31	Sakuranetin	22.38	tr	4.45	6.95	16.36	1.70	10.03	17.08
32	Galangin	22.43	tr	tr	16.67	tr	tr	tr	tr
33	*Pinobanksin-7-methyl ether^b^	22.62	nd	tr	10.78	0.17	3.43	tr	1.61
34	2-Acetyl-1,3-di-*p*-coumaroylglycerol^g^	22.72	0.64	nd	nd	2.01	tr	13.04	tr
35	Pinobanksin-3-*O*-acetate^b^	22.80	tr	tr	43.92	tr	1.11	tr	0.99
36	Kaempferide (4′-methylkaempferol)^h^	22.93	tr	0.82	1.48	1.07	tr	tr	1.85
37	*p*-Coumaric acid prenyl or isoprenyl ester I^g^	23.11	nd	tr	1.57	0.35	0.26	nd	0.08
38	Methoxychrysin^c^	23.21	nd	tr	3.87	tr	tr	nd	0.06
39	*p*-Coumaric acid prenyl or isoprenyl ester II^g^	23.38	nd	tr	2.88	0.09	0.48	0.45	tr
40	*p*-Coumaric acid benzyl ester^g^	23.88	0.35	nd	1.35	0.81	tr	8.17	0.08
41	Ferulic acid benzyl ester^i^	24.65	0.33	nd	3.09	0.61	0.40	4.78	0.40
42	Pinobanksin 3-*O*-propanoate^b^	25.05	nd	tr	6.50	tr	0.88	tr	0.34
43	Myricetin-3,7,4′,5′-tetramethyl-ether^f^	25.11	4.29	nd	nd	nd	nd	nd	nd
44	*p*-Coumaric acid cinnamyl ester^g^	26.83	nd	tr	1.65	tr	tr	tr	tr
45	Pinobanksin 3-*O*-pentenoate or isopentenoate^b^	27.06	nd	nd	15.70	nd	tr	tr	2.85
46	Pinostrobin	27.20	nd	tr	tr	0.50	tr	tr	4.16

* Component tentatively identified; ^a^ calculated as caffeic acid equivalent; ^b^ calculated as pinobanksin equivalent; ^c^ calculated as chrysin equivalent; ^d^ calculated as luteolin equivalent; ^e^ calculated as galangin equivalent; ^f^ calculated as quercetin equivalent; ^g^ calculated as *p*-coumaric acid equivalent; ^h^ calculated as kaempferol equivalent; ^i^ calculated as ferulic acid equivalent.

**Table 5:** Total phenol, flavonoid content and antioxidant properties of the propolis samples.

	TP ^a^		TF ^b^		DPPH ^c^		FRAP ^d^	
Propolis	[mg GAE/g]	±SD	[mg QE/g]	±SD	[mg GAE/g]	±SD	[mmol Fe^2+^/g]	±SD
BP	14.0	0.9	8.8	0.1	2.6	0.1	0.1	0.0
KP	15.9	0.9	11.1	0.3	2.7	0.1	0.2	0.0
RP	189.7	1.5	103.9	4.2	81.6	5.2	0.8	0.0
PP	22.5	1.2	11.1	0.2	6.3	0.2	0.3	0.0
K1P	36.7	1.8	14.9	0.3	31.8	2.2	0.5	0.1
K2P	33.2	0.3	7.2	0.4	11.2	0.2	0.4	0.0
K3P	26.1	0.8	18.0	0.6	12.7	0.4	0.4	0.0

Data are expressed as average of 3 measurements ± standard deviation (SD); ^a^ Total phenolics (TP) value is expressed as gallic acid equivalent (GAE). ^b^ Total flavoniods (TF) value is expressed as quercetin equivalent (QE) ^c^ DPPH value is expressed as gallic acid equivalent (GAE) having an equivalent antiradical capacity. ^d^ FRAP value is expressed as millimolar concentration of Fe^2+^, obtained from a dilution of FeSO_4_ having an equivalent antioxidant capacity.
